# High salt diet accelerates the progression of murine lupus through dendritic cells *via* the p38 MAPK and STAT1 signaling pathways

**DOI:** 10.1038/s41392-020-0139-5

**Published:** 2020-04-10

**Authors:** Ze Xiu Xiao, Xiaojiang Hu, Ximei Zhang, Zhigang Chen, Julie Wang, Ke Jin, Feng Lin Cao, Baoqing Sun, Joseph A. Bellanti, Nancy Olsen, Song Guo Zheng

**Affiliations:** 1 Department of Clinical Immunology, The Third Affiliated Hospital at Sun Yat-sen University, 510630 Guangzhou, China; 2 Department of Internal Medicine, Ohio State University College of Medicine and Wexner Medical Center, Columbus, OH 43210 USA; 3 Department of Medicine, Penn State College of Medicine, Hershey, PA 17033 USA; 4 Laboratory of Human Diseases and Immunotherapy, West China Hospital, Sichuan University, Chengdu, China; 5 Department of Internal Medicine, The First Hospital of Harbin Medical University, Harbin, China; 6 Department of Allergy and Clinical Immunology, The First Affiliated Hospital of Guangzhou Medical University, Guangzhou, China; 7 Department of Pediatrics and Microbiology-Immunology, Georgetown University Medical Center, Washington, DC USA

**Keywords:** Rheumatology, Immunology

## Abstract

The increased incidence of systemic lupus erythematosus (SLE) in recent decades might be related to changes in modern dietary habits. Since sodium chloride (NaCl) promotes pathogenic T cell responses, we hypothesize that excessive salt intake contributes to the increased incidence of autoimmune diseases, including SLE. Given the importance of dendritic cells (DCs) in the pathogenesis of SLE, we explored the influence of an excessive sodium chloride diet on DCs in a murine SLE model. We used an induced lupus model in which bone marrow-derived dendritic cells (BMDCs) were incubated with activated lymphocyte-derived DNA (ALD-DNA) and transferred into C57BL/6 recipient mice. We observed that a high-salt diet (HSD) markedly exacerbated lupus progression, which was accompanied by increased DC activation. NaCl treatment also stimulated the maturation, activation and antigen-presenting ability of DCs in vitro. Pretreatment of BMDCs with NaCl also exacerbated BMDC-ALD-DNA-induced lupus. These mice had increased production of autoantibodies and proinflammatory cytokines, more pronounced splenomegaly and lymphadenopathy, and enhanced pathological renal lesions. The p38 MAPK–STAT1 pathway played an important role in NaCl-induced DC immune activities. Taken together, our results demonstrate that HSD intake promotes immune activation of DCs through the p38 MAPK–STAT1 signaling pathway and exacerbates the features of SLE. Thus, changes in diet may provide a novel strategy for the prevention or amelioration of lupus or other autoimmune diseases.

## Introduction

Systemic lupus erythematosus (SLE) is a chronic inflammatory autoimmune disease that is characterized by increased production of various autoantibodies against autoantigens and mainly affects women of childbearing age.^1–4^ Although the etiology of SLE is still incompletely understood, genetic susceptibility, hormonal modulation, environmental factors, and gut microbes related to diet are involved in the development of SLE.^5–8^ Abnormalities in the adaptive immune system, including B and T cells and the production of pathogenic autoantibodies,^9–14^ and components of the innate immune system, including dendritic cells and complement, all participate in the occurrence and development of SLE.^15–18^ SLE pathogenic inflammatory T cells contribute to cytokine generation and help abnormal B cells produce autoantibodies.^19^ Autoantibodies and complement deposition in kidneys and in other organs cause irreversible damage.^20–22^ Therefore, the treatment of lupus remains unsatisfactory,^23–26^ even with some of the newer biologics.^27^ Approaches to modifying diet may have the potential to significantly improve disease management.

Recently, increased sodium chloride (NaCl) intake has been shown to promote autoimmune disease in an experimental autoimmune encephalomyelitis model either by increasing Th17 cell function^28–31^ or by promoting proinflammatory macrophage polarization and exacerbation of central nervous system (CNS) autoimmunity.^32^ High NaCl treatment also promotes autoimmunity by TET2-induced DNA demethylation and differentiation of Tfh (follicular T helper) cells, thus accelerating the development of lupus syndromes in an experimental MRL/lpr mouse model^33^ and demonstrating the critical role of Tfh cells in the production of autoantibodies and pathogenesis of lupus.^34–38^

Dendritic cells are professional antigen-presenting cells that present antigen to T cells and initiate adaptive immunity.^39–41^ CD11c is an ideal surface marker of murine dendritic cells.^42^ Dendritic cells are mediators between the innate and adaptive immune systems, linking the two systems in a bidirectional and interdependent manner.^43,44^ Moreover, dysfunction of dendritic cells contributes to the development of autoimmune diseases such as type I diabetes and multiple sclerosis.^45–47^ Dendritic cells also participate in the initiation and development of SLE.^48,49^ Previously, we established a murine lupus model by transferring bone marrow-derived dendritic cells (BMDCs) that had been incubated with activated lymphocyte-derived DNA (ALD-DNA) into C57BL/6 or Balb/c mice,^50^ resulting in a specific dendritic cell-mediated lupus model for the proposed study.^51–55^ Anti-dsDNA antibodies and lupus-like kidney pathological changes, including proteinuria and glomerulonephritis with hyperplasia in the glomeruli, increased mesangial cell numbers and vasculitis with perivascular cell infiltration, and glomerular deposition of IgG and complement C3, are characteristic manifestations of this model.^50,56^ Using this model, we aimed to determine whether sodium chloride exacerbates lupus syndrome in BMDC-ALD-DNA-induced lupus mice and to investigate whether sodium chloride has the effect on dendritic cells.

Our results demonstrate that sodium chloride markedly promotes the activation and maturation of dendritic cells. These dendritic cells were pretreated with sodium chloride, incubated with ALD-DNA, and then transferred into mice to induce lupus-like syndromes. These dendritic cells were characterized by increased expression of antigen presentation-associated markers and by enhanced proinflammatory cytokine production. We further found that sodium chloride promoted p38 MAPK–STAT1 signaling in dendritic cells. These results suggest that an HSD accelerates lupus pathogenesis by enhancing the differentiation and function of inflammatory dendritic cells. Thus, normal or low-salt intake might be recommended to patients with SLE and may contribute to reduced incidence of flares and better therapeutic effects and prognosis of SLE.

## Results

### Sodium chloride-rich diet promotes BMDC-ALD-DNA-induced lupus and NZM2328 lupus accompanied by increased activation of dendritic cells

**Fig. 1:**
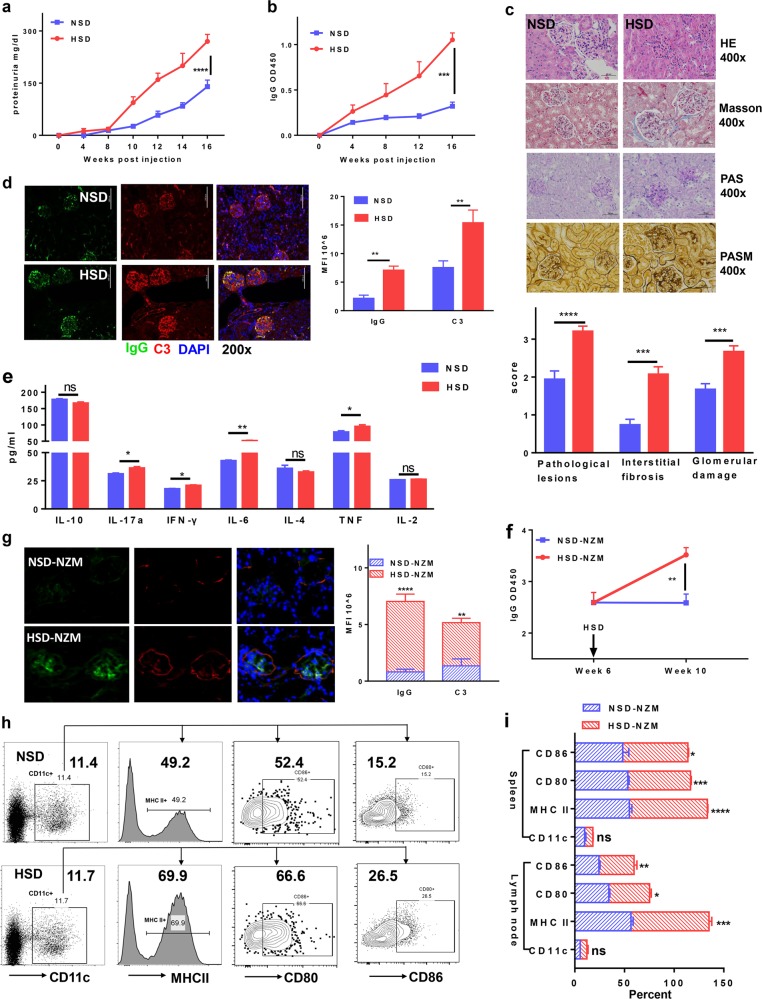
A high-salt diet enhanced lupus in a bone marrow cell-derived dendritic cell-ALD-DNA-induced murine lupus model and in NZM2328 lupus mice. **a–e** Bone morrow-derived dendritic cells (0.5 × 10^6^) were incubated with ALD-DNA and intravenously transferred to normal C57BL/6 mice that were fed either a normal-salt diet (NSD) or a high-salt diet (HSD) (*n* = 5 in one individual experiment). **a** Total serum IgG anti-dsDNA was measured and compared between the HSD and NSD groups. **b** Proteinuria levels between the HSD and NSD groups. **c** H&E, Masson, periodic acid-Schiff (PAS), and periodic acid-silver methenamine (PASM) staining of renal sections and the pathological scores of lupus nephritis of the HSD and NSD lupus mice at 18 weeks after BMDC-ALD-DNA transfer into the recipient mice; pathological lesion scores represent HE staining, the interstitial fibrosis scores represent Masson staining, and the glomerular damage score represents periodic acid-Schiff (PAS) staining of the kidney sections. **d** Immunofluorescence showing the deposition of IgG and C3 in renal sections from HSD lupus mice and NSD lupus mice. **e** A CBA kit was used for quantitative measurement of cytokines in sera from HSD lupus mice compared with those of NSD lupus mice. **f**, **g** Six-week-old NZM2328 mice received a high-salt diet or a control normal-salt diet for 4 weeks (*n* = 4 in each experiment). **f** ELISA semiquantitative measurement of total IgG against dsDNA in sera from HSD-treated mice compared with that of NSD-treated NZM2328 mice. **g** Immunofluorescence showing the deposition of IgG and C3 in the renal sections from the HSD mice compared with that of NSD NZM2328 mice. **h**, **i** Flow cytometry analysis of the dendritic cell ratios and activation markers, including MHC II, CD80, and CD86, in the spleen from NSD BMDC-ALD-DNA-induced lupus mice and HSD BMDC-ALD-DNA-induced lupus mice (**h**) or spontaneous lupus NZM2328 mice with HSD or NSD (**i**). The results are presented as the mean ± s.e.m. from three separate experiments. ns indicates no significance, **p < 0.005, ***p < 0.0005, and ****p < 0.0001 using nonparametric Mann–Whitney tests. Scale bars: ×200: 100 μm, ×400: 50 μm.

To test the hypothesis that high salt promotes lupus by affecting dendritic cell function, we used the BMDC-ALD-DNA-induced lupus murine model that has a DC-induced lupus-like syndrome. We previously established that BMDC-ALD-DNA induces murine SLE through dendritic cell-specific initiation.^50,56^ We observed that a diet enriched in sodium chloride promoted murine lupus disease. The levels of anti-dsDNA antibodies (OD450 of IgG in NSD: 0.3198 ± 0.04588 vs HSD: 1.053 ± 0.07519) (Fig. 1a, Supplementary Fig. 1a) and proteinuria (NSD: 210 ± 36.74 mg/dl vs HSD: 360 ± 29.15 mg/dl) (Fig. 1b) were increased in induced lupus mice with excessive sodium chloride intake (HSD lupus mice) compared to those of the lupus model with normal sodium chloride intake (NSD lupus mice) (IgG: *p* value <0.001; proteinuria: *p* value = 0.0127). The HSD lupus mice also displayed marked exacerbation of pathologic manifestations of lupus nephritis. Using H&E, Masson, periodic acid-Schiff (PAS), and periodic acid-silver methenamine (PASM) staining of lupus mouse kidney paraffin sections, severe renal pathological lesions were more pronounced in kidneys from HSD lupus mice than in those from NSD lupus mice (Fig. 1c). Similarly, the deposition of immunoglobulin and complement C3 in kidney lesions was more pronounced in HSD lupus mice than in NSD mice (Fig. 1d). Consistent with these alterations, the proinflammatory cytokines IL-17a, IFN-γ, IL-6, and TNF in sera were also higher HSD mice than in NSD control mice (Fig. 1e, Table 1). Splenomegaly and lymphadenopathy were also more pronounced in HSD mice than in NSD mice (Supplementary Fig. 1b).

**Table 1:** Cytokines in serum from two groups: BMDC-ALD-DNA-induced lupus mice in normal-salt diet (NSD) and high-salt diet (HSD).

Group^a^	IL-17a	IFN-γ	IL-6	TNF
NSD	30.92 ± 1.048	17.57 ± 0.5997	42.67 ± 0.7765	74.83 ± 4.163
HSD	36.06 ± 1.542	20.64 ± 0.7472	50.79 ± 2.028	97.27 ± 5.286
*P* value	0.0328	0.0297	0.0144	0.0157

CBA kit quantitative of cytokines in sera from the HSD lupus mice compared with NSD lupus mice. The results are displayed as the mean (±s.e.m.) from three independent experiments.

^a^The unit is pg/ml.

To further investigate whether an HSD exacerbates lupus development, we used an additional lupus model, NZM2328, to further address this possibility. NZM2328 is a spontaneous SLE-prone murine strain that has been extensively used in lupus research.^57–59^ We found that a sodium chloride-rich diet increased the level of anti-dsDNA autoantibodies in NZM2328 mice (Fig. 1f), as well as the pathological changes in lupus nephritis, as manifested by IgG and C3 deposition (Fig. 1g).

Since dendritic cells are the key drivers of ALD-DNA-induced lupus,^50,56^ a separate set of experiments was performed to determine whether high sodium chloride promotes lupus through stimulation of dendritic cells. Although the numbers or ratios of dendritic cells in spleens (Fig. 1h) or peripheral blood (data not shown) showed no differences between NSD and HSD lupus mice, the activation markers (MHC II, CD80, and CD86) on dendritic cells were significantly higher in HSD lupus mice than in NSD lupus mice. Moreover, we also noted that the activation markers (MHC II, CD80, and CD86) on dendritic cells were significantly elevated in spontaneous lupus NZM2328 mice that were fed the HSD diet compared with those that were fed the NSD diet (Fig. 1i). Although the DC population has different subsets and surface molecular markers, CD11c is one of most specific markers for DCs.^60^ Because a small population of neutrophils express CD11c, we also examined the frequency of neutrophils by flow cytometry under the HSD or NSD and found that the HSD did not affect the frequency of neutrophils (Supplementary Fig. 2). Thus, we believe that the promotion of murine lupus by high sodium chloride intake was accompanied by increased activation of dendritic cells.

The effect of excessive sodium chloride intake on other immune cells in the induced lupus model was also investigated. B cells (B220+), plasma cells (CD38+ CD138+), CD4+ T cells, Tfh cells (follicular T help cells, CD4+ PD-1+ CXCR5+), GCB cells (germinal center B cells, CD4-B220+ IgD-GL7+^61^, or CD4-B220+ GL7+CD95+^62–64^), IL-17a+ T cells, and IFN-γ+ T cells were all increased in HSD lupus mice compared to those in NSD lupus mice (Supplementary Fig. 3). The frequency and levels of the activation marker OX40 on Tfh cells were significantly increased in HSD lupus mice compared to those in NSD lupus mice. Conversely, the inactivation marker CD62L was reduced on Tfh cells from HSD lupus mice compared with those of NSD lupus mice (Supplementary Fig. 3a). Since Treg cells play a crucial role in the prevention of autoimmune diseases, including lupus,^65–69^ and an HSD affects thymus-derived natural Treg (tTreg) cells,^70^ surprisingly, Foxp3+ CD4+ regulatory T cells, including induced Treg (iTreg) and nTreg cell subsets, showed no differences between the two lupus groups (Supplementary Fig. 3c).

We also tried to exclude other possibilities to explain how an HSD contributes to the development of lupus beyond DC activation. These were as follows: (1) inflammation and lupus-like syndromes are caused by apoptosis and death of the injected DCs; and (2) HSD also increases fluid intake and directly affects kidney function. We first developed an in vivo imaging experiment to clearly show that these injected DCs were distributed in various organs (Supplementary Fig. 4a) and that these cells were less apoptotic at 7 days (Supplementary Fig. 4b) and 14 days (data not shown) after DC transfer. Furthermore, we used CD45.1 C57BL/6 mouse-derived BMDCs that were incubated with or without ALD-DNA and injected these cells into CD45.2 C57BL/6 mice; at 14 days after injection, we found that the donor dendritic cells that had been incubated with ALD-DNA exerted higher activation than those that had not been incubated with ALD-DNA, while the recipient dendritic cells showed no differences (Supplementary Fig. 5). We measured and compared the fluid intake of mice and correlated it with lupus syndromes with or without an HSD. The fluid intake of lupus mice that were fed an HSD was nearly threefold greater than that of lupus mice that were fed a normal diet (17.5 vs 6 ml per mouse per day). We also chose B6 WT mice as a control. Similarly, the fluid intake of B6 WT mice that were fed an HSD was also threefold greater than that of normal diet B6 WT mice; however, there were no detectable levels of anti-dsDNA antibodies, proteinuria, or renal pathology in mice that were fed an HSD during this period (data not shown). Thus, we believe that the inflammation in mice was not attributed to DC death and that the HSD promoted lupus mainly by affecting immune cells but not by increasing fluid intake, which indirectly influenced kidney function.

### Sodium chloride does not stimulate dendritic cell differentiation but accelerates activation in vitro

**Fig. 2:**
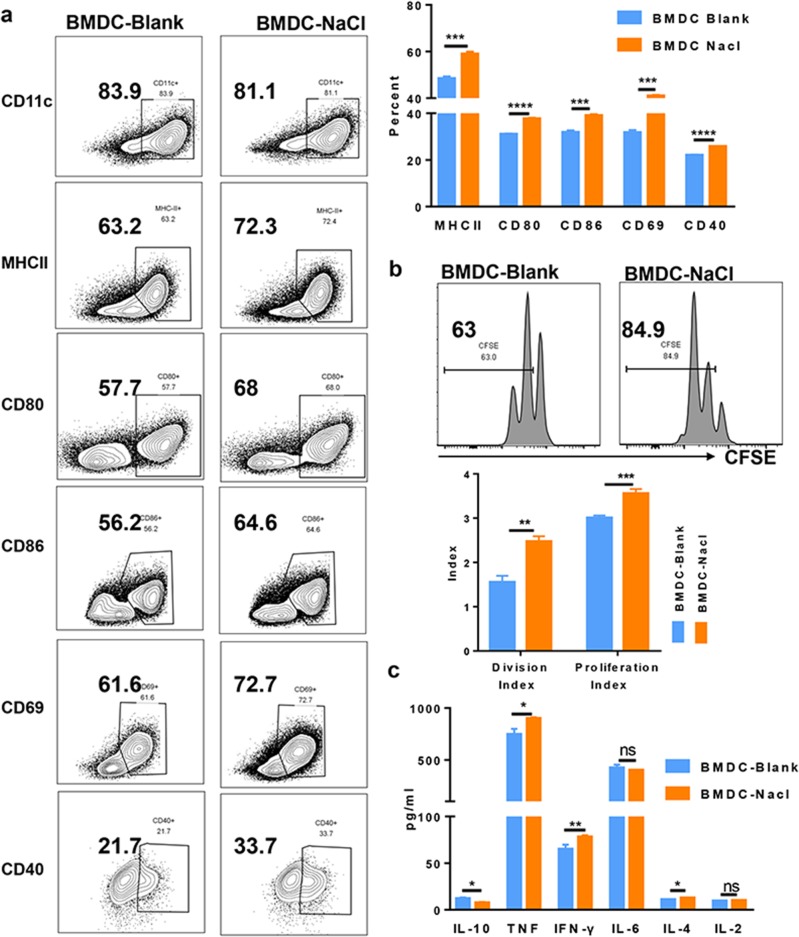
NaCl accelerated activation of bone marrow cell-derived dendritic cells in vitro. **a** Sodium chloride (20 mM) or control medium was added to the cultures of ALD-DNA-DCs for 24 h, and then the activation and maturation status of dendritic cells was analyzed by flow cytometry to compare the two groups. **b**, **c** Bone marrow-derived dendritic cells from C57BL/6 mice were pretreated with 10 ng/ml LPS and/or 20 mM NaCl for 24 h. These cells were collected for coculture with CFSE-labeled T cells from Balb/c mice for mixed lymphocyte reactions. Flow cytometry analysis of CFSE, which indicates T cell proliferation and indirectly represents the antigen-presenting ability of dendritic cells (**b**). A CBA cytokine kit was used to measure mediators in the supernatants after DC-T cell coculture for 72 h (**c**). The results are presented as the mean ± s.e.m. from three independent experiments. ns indicates no significance, **p < 0.005, ***p < 0.0005, and ****p < 0.0001 using paired Student’s *t*-tests.

Excessive sodium chloride dietary intake promotes the activation and maturation of dendritic cells in an in vivo lupus model; however, we do not know whether high sodium chloride first activates DCs and then promotes lupus. Using a standard in vitro protocol for dendritic cell differentiation from bone marrow precursors,^71,72^ we next sought to determine whether sodium chloride directly affects the differentiation, activation, and maturation of dendritic cells. Following the addition of sodium chloride (20 mM) to dendritic cell cultures, the numbers of dendritic cells did not increase compared to those of control cultures (data not shown). As culture medium contains some amounts of NaCl (6 g/L, 100 mM), after adding 20 mM NaCl to the medium, the final concentration of NaCl was 149 mM. This concentration was used for in vitro cell culture, since it increases T cell activity but does not reduce T cell viability.^31,70^ We also demonstrated that the addition of 20 mM NaCl did not affect BMDC apoptosis or viability (Supplementary Fig. 6a). However, dendritic cells showed increased activation after stimulation with ALD-DNA compared with that of the control treatment. The activation markers included MHC II, CD80, and CD86, which is consistent with the results reported previously.^73^ Additionally, CD69 and CD40 were also significantly increased when sodium chloride was added (Fig. 2a). We next assessed dendritic cell function following sodium chloride treatment. Dendritic cells present antigens to T cells and then initiate the activation and proliferation of T cells. Using a previously described allogeneic mixed lymphocyte cell culture system,^74^ sodium chloride-pretreated dendritic cells induced a significant increase in T cell proliferation (Fig. 2b). In addition, dendritic cells secrete various cytokines to helper T cells. Using the multi-cytokine CBA assay kit, we found that proinflammatory cytokines such as TNF, IFN-γ, and IL-4 were more highly secreted from dendritic cells that were pretreated with sodium chloride compared to those of control cultures that were treated with LPS, while the anti-inflammatory cytokine IL-10 showed the opposite changes and was secreted at lower concentrations in salt-treated cultures than in controls (TNF: *p* value = 0.0140; IFN-γ: *p* value = 0.0041; IL-4: *p* value = 0.0439; IL-10: *p* value = 0.0128) (Fig. 2c). Thus, sodium chloride accelerated the activation and antigen-presenting ability and altered cytokine secretion of the dendritic cells, resulting in increased immune reactivity and lupus disease progression.

### Pretreatment of BMDCs with sodium chloride accelerated the murine lupus model

**Fig. 3:**
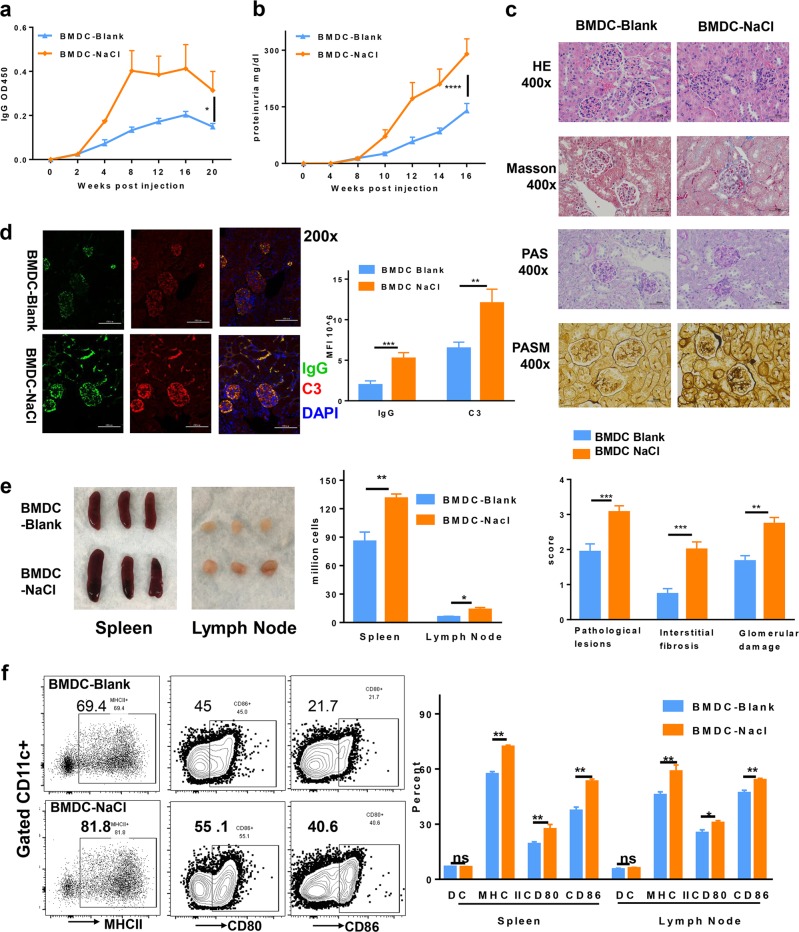
Bone marrow-derived dendritic cells that were pretreated with NaCl accelerated the murine lupus model. Bone morrow-derived dendritic cells were incubated with ALD-DNA (0.5 × 10^6^) with or without NaCl (20 mM) and were intravenously transferred to normal C57BL/6 mice (*n* = 5 in one individual experiment). **a** ELISA semiquantitative measurement of total IgG against dsDNA in sera from NaCl-pretreated and control-pretreated BMDC-ALD-DNA-induced lupus mice. **b** Proteinuria levels of NaCl-pretreated and control-pretreated BMDC-ALD-DNA-induced lupus mice. **c** H&E, Masson’s trichrome, periodic acid-Schiff (PAS), and periodic acid-silver methenamine (PASM) staining of renal sections from the NaCl-pretreated and control-pretreated BMDC-ALD-DNA-induced lupus mice 18 weeks after cell transfer. The pathological lesion scores represent HE staining, the interstitial fibrosis scores represent Masson staining, and the glomerular damage score represents periodic acid-Schiff (PAS) staining of the kidney sections. **d** Immunofluorescence staining showing the deposition of IgG and C3 in renal sections from NaCl-pretreated and control-pretreated BMDC-ALD-DNA-induced lupus mice. **e** The gross appearance of and the total cell number in the spleens or lymph nodes from NaCl-pretreated BMDC-ALD-DNA-induced lupus mice compared with those of control BMDC-ALD-DNA-induced lupus mice. **f** Flow cytometry analysis showing the dendritic cell ratios and activation markers, including MHC II, CD80, and CD86, in the spleens and lymph nodes of NaCl-pretreated and control-pretreated BMDC-ALD-DNA-induced lupus mice. The results are presented as the mean ± s.e.m. from three separate experiments. ns indicates no significance, **p < 0.005, ***p < 0.0005, and ****p < 0.0001 using nonparametric Mann–Whitney tests. Scale bars: ×200: 100 μm, ×400: 50 μm.

The functional characteristics of dendritic cells that were pretreated with sodium chloride were next evaluated in the induced lupus model. Prior to transfer into mice, BMDCs were pretreated with control medium or 20 mM NaCl and then incubated with ALD-DNA. Consistent with our ex vivo findings, adoptive transfer of sodium chloride-pretreated BMDC-ALD-DNA induced more severe lupus symptoms than that of infusion of control BMDC-ALD-DNA in induced lupus mice; the ratio of dendritic cells was similar between the control and NaCl pretreatment groups (Supplementary Fig. 6b). The levels of anti-dsDNA antibodies (Fig. 3a, Supplementary Fig. 7a) (IgG: BMDC-Blank 0.1488 ± 0.01504 vs BMDC-NaCl 0.3138 ± 0.08621) and proteinuria (Fig. 3b) (BMDC-Blank 200 ± 27.39 mg/dl vs BMDC-NaCl 360 ± 24.49 mg/dl) were markedly increased in the sodium chloride-pretreated BMDC-ALD-DNA lupus group (IgG: *p* value = 0.0435; proteinuria: *p* value = 0.0024). Moreover, the renal pathological lesions were more severe in sodium chloride-pretreated BMDC-ALD-DNA-induced lupus mice (Fig. 3c). Immunofluorescence staining in the kidney revealed that IgG and C3 immune deposition in the glomeruli and interstitium was similarly increased in the sodium chloride group compared to that of control-pretreated BMDC-ALD-DNA-induced lupus mice (Fig. 3d). Serum proinflammatory cytokines, including IL-17a, IFN-γ, IL-6, TNF, and IL-2, were significantly increased, while the anti-inflammatory cytokine IL-10 was significantly decreased in sodium chloride-pretreated BMDC-ALD-DNA-induced lupus mice (Supplementary Fig. 7b, Table 2). The lymphatic organs from sodium chloride-pretreated BMDC-ALD-DNA-induced lupus mice displayed more severe splenomegaly and lymph node enlargement than those of the control BMDC-ALD-DNA-induced lupus mice (Fig. 3e). Proinflammatory immune cells, including B cells, plasma cells, CD4+ T cells, Tfh cells, GCB cells, IL-17a+ T cells, and IFN-γ+ T cells, were increased in sodium chloride-pretreated BMDC-ALD-DNA-induced lupus mice compared to those in control BMDC-ALD-DNA-induced lupus mice (Supplementary Fig. 8). Furthermore, OX40 expression was increased in Tfh cells, and CD62L expression was reduced in Tfh cells from sodium chloride-pretreated BMDC-ALD-DNA-induced lupus mice (Supplementary Fig. 8a). Regulatory T cells, including iTreg and nTreg subsets, showed no differences between the two groups of mice (Supplementary Fig. 8c). The expression of Helios and Nrp1 was used to distinguish iTreg and nTreg cells.^75–77^ Interestingly, dendritic cell ratios in the spleens and draining lymph nodes showed no differences between the two groups of lupus mice. However, the maturation and activation markers on dendritic cells, including MHC II, CD80, and CD86, were significantly increased in cells that were isolated from sodium chloride-pretreated BMDC-ALD-DNA-induced lupus mice compared to those of control lupus mice (Fig. 3f). Based on these results, we suggest that pretreatment of BMDCs with sodium chloride increased their activation and maturation, accelerating the development of lupus in the murine lupus model.

**Table 2:** Cytokines in serum from two groups: BMDC-Blank and BMDC-NaCl-induced lupus mice.

Group^a^	IL-17a	IFN-γ	IL-6	TNF	IL-2	IL-10
BMDC-Blank	26.9 ± 0.782	17.23 ± 0.544	39.38 ± 2.202	75.53 ± 2.92	26.49 ± 0.5706	178.6 ± 2.387
BMDC-NaCl	32.99 ± 1.408	24.4 ± 0.2081	54.8 ± 1.977	94.4 ± 3.928	32.7 ± 1.488	149.6 ± 10.05
*P* value	0.0020	<0.0001	0.0003	0.0032	0.0046	0.0186

CBA kit quantitative of cytokines in sera from the NaCl-pretreated BMDCs-ALD-DNA-induced lupus mice compared with control BMDCs-ALD-DNA-induced lupus mice. The results are displayed as the mean (±s.e.m.) from three independent experiments.

^a^The unit is pg/ml.

### Sodium chloride-pretreated BMDCs increase the severity of lupus features via the p38 MAPK–STAT1 pathway

**Fig. 4:**
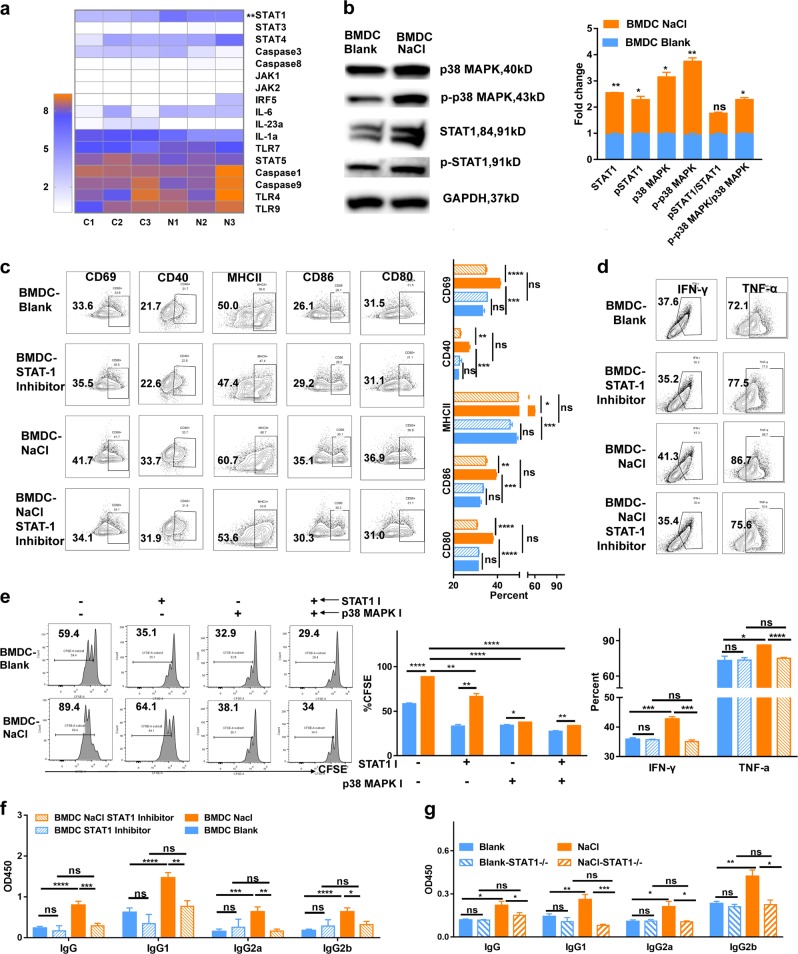
NaCl facilitated the function of dendritic cells through the p38 MAPK–STAT1 pathway. Bone morrow-derived dendritic cells were incubated with ALD-DNA with or without NaCl (20 mM) and were treated with STAT1 inhibitor (2 μg/ml), p38 MAPK inhibitor (5 μM), or control (DMSO). **a** Quantitative RT-PCR array showing the control-treated BMDCs and NaCl-treated BMDCs; C1, C2, or C3 represents control-treated BMDCs and N1, N2, or N3 represents NaCl-treated BMDCs. STAT1 expression was the only parameter to show a statistically significant difference (*p* value = 0.0034). **b** Total STAT1, p38 MAPK, phosphorylated STAT1, and phosphorylated p38 MAPK in control-treated BMDCs and NaCl-treated BMDCs. **c** Flow cytometry analysis showing the activation and maturation markers in BMDCs with or without NaCl treatment in the presence or absence of the STAT1 inhibitor. **d** Flow cytometry analysis showing IFN-γ and TNF-α expression in BMDCs with or without NaCl treatment in the presence or absence of the STAT1 inhibitor. **e** Bone marrow-derived dendritic cells from C57BL/6 mice were pretreated with 10 ng/ml LPS and/or 20 mM NaCl and/or 2 μg/ml STAT1 inhibitor and/or 5 μM p38 MAPK inhibitor for 24 h and then were collected for coculture with CFSE-labeled T cells from Balb/c mice for 72 h; the CFSE in T cells was analyzed. **f** Bone marrow-derived dendritic cells were incubated ALD-DNA with or without NaCl treatment (20 mM) in the presence or absence of 2 μg/ml STAT1 inhibitor and were then transferred to normal C57BL/6 mice (*n* = 5 in each experiment). ELISA results showing semiquantitative measurement of IgG, IgG1, IgG2a, and IgG2b anti-dsDNA in sera from each group of lupus mice 4 weeks after BMDC-ALD-DNA injection. **g** Bone marrow-derived dendritic cells from wild type and STAT1^−/−^ mice were incubated with ALD-DNA with or without NaCl treatment (20 mM) were then transferred to normal C57BL/6 mice (*n* = 5 in each individual experiment). ELISA measurements showing IgG, IgG1, IgG2a, and IgG2b anti-dsDNA in sera from each group of lupus mice 8 days after BMDC-ALD-DNA injection. The results are presented as the mean ± s.e.m. from three independent experiments. ns indicates no significance, **p < 0.005, ***p < 0.0005, and ****p < 0.0001 using paired Student’s *t*-tests or nonparametric Mann–Whitney tests.

We next sought to investigate the molecular basis for the activation and enhanced functional effects of high sodium chloride on dendritic cells. We first used a quantitative RT-PCR array to investigate the involvement of different factors in BMDCs treated with and without sodium chloride. Interestingly, we observed that the transcription level of STAT1 increased significantly in sodium chloride-treated BMDCs (Fig. 4a). Since p38 MAPK kinase is required for interferon-induced STAT1 serine phosphorylation and transcriptional activation,^78^ we examined the p38 MAPK and STAT1 protein levels in BMDCs and found that p38 MAPK and STAT1 were significantly increased in dendritic cells that were treated with sodium chloride; otherwise, the ratio of phospho-STAT1/total STAT1 showed no differences between blank BMDCs and NaCl-treated BMDCs, while the ratio of phospho-p38 MAPK/total p38 MAPK increased in NaCl-treated BMDCs compared with that of blank BMDCs (Fig. 4b). The increased expression of activation and maturation markers in dendritic cells, including CD80, CD86, MHC II, CD40, and CD69, was significantly reduced when a specific STAT1 inhibitor or p38 MAPK inhibitor was added to the cultures (Fig. 4c, Supplementary Fig. 9). Moreover, the mediators IFN-γ and TNF-α were significantly increased when sodium chloride was added to BMDCs that were stimulated with ALD-DNA and were decreased when the STAT1 inhibitor was used (Fig. 4d). STAT1 knockout BMDCs (BMDC^STAT1−/−^) showed no differences in activation or maturation when sodium chloride was added compared with those of the control blank (Supplementary Fig. 10). The increased antigen-presenting capacity of dendritic cells to drive the proliferation of T cells with sodium chloride treatment was partly diminished when STAT1 or p38 MAPK was inhibited (Fig. 4e), in addition to the capacity of dendritic cells to drive T cells to secrete IL-17 (Supplementary Fig. 11). To investigate these findings further in the in vivo lupus model, we next sought to validate whether sodium chloride promoted dendritic cell activation and exacerbated lupus through activation of the p38 MAPK–STAT1 pathway in dendritic cells. In this experiment, BMDCs were treated with a STAT1 inhibitor or p38 MAPK with or without sodium chloride, and then BMDC-ALD-DNA was transferred into normal mice to induce lupus under a normal-salt diet (NSD). We observed that the increased levels of autoantibodies in the lupus mice were significantly reduced in dendritic cells that were pretreated with the STAT1 inhibitor or p38 MAPK inhibitor compared to those that were pretreated with the corresponding control (Fig. 4f, Table 3, Supplementary Fig. 12). Furthermore, NaCl pretreatment of BMDC^STAT1−/−^-ALD-DNA did not induce an increased level of anti-dsDNA antibodies compared with that of Blank BMDC^STAT1−/−^-ALD-DNA (Fig. 4g). Thus, these results indicate that sodium chloride treatment promotes the activation and maturation of dendritic cells through p38 MAPK–STAT1 signaling, resulting in an exacerbation of lupus.

**Table 3:** Anti-dsDNA antibodies levels from four groups: BMDC-Blank, BMDC-STAT1 inhibitor, BMDC-NaCl, BMDC-NaCl-STAT1 inhibitor-induced lupus mice.

Group^a^	BMDC pretreatment		Antibody subsets			
	NaCl	STAT1 inhibitor	IgG	IgG1	IgG2a	IgG2b
BMDC-Blank	–	–	0.2345 ± 0.04439	0.6189 ± 0.1101	0.1494 ± 0.06138	0.1736 ± 0.03556
BMDC-STAT1 inhibitor	–	+	0.4452 ± 0.1462	0.8842 ± 0.2671	0.4658 ± 0.2375	0.2914 ± 0.1605
BMDC-NaCl	+	–	0.7961 ± 0.09488	1.466 ± 0.128	0.632 ± 0.1225	0.6318 ± 0.09957
BMDC-NaCl-STAT1 inhibitor	+	+	0.2913 ± 0.05925	0.769 ± 0.137	0.1624 ± 0.04777	0.3222 ± 0.07779

The presenting data were OD levels represent the anti-dsDNA antibodies levels from grouping mice. Bone morrow-derived dendritic cells incubated ALD-DNA with or without treatment of NaCl (20 mM) in the presence or absence of 2 μM STAT1 inhibitor and were then transferred to normal C57BL/6 mouse (*n* = 5 in each individual experiment). ELISA experiment semiquantitative of total IgG against dsDNA in sera from each group lupus mice. The results are displayed as the mean (±s.e.m.) from three independent experiments.

^a^The data represent OD_450_.

## Discussion

Accumulating evidence supports a role for the contribution of environmental factors to the increase in immune disorders, including autoimmune diseases and chronic inflammatory diseases. High-salt intake promotes the development of Th17 cells through the MAPK/p30 pathway, thereby exacerbating experimental autoimmune encephalomyelitis (EAE).^79^ Related studies have also shown that sodium chloride promotes proinflammatory macrophage polarization by altering MAPK signaling in macrophages and thus facilitating CNS autoimmunity.^32^ Our recent study demonstrated that high-salt intake promotes colitis by enhancing Th17 cell differentiation and expansion, although there were different impacts on different Treg cell subsets.^31^ Since the influence of sodium chloride on dendritic cells has not been fully explored, a major investigative effort of the present study focused on the specific effect of sodium chloride on dendritic cells using a lupus model; this approach was supported by the results of our previous study that used BMDCs that were incubated with ALD-DNA and transferred to mice by tail vein injection.^50,56^ This experimental murine lupus model provides a suitable system for investigating the role of dendritic cells in autoimmune disease.

In the present study, we first demonstrated that a salt-rich diet facilitated the activation and maturation of dendritic cells and exacerbated murine lupus. Salt-rich dietary intake also increased the proinflammatory features of dendritic cells in lupus-induced mice. Although excessive salt did not increase the number of dendritic cells, it significantly promoted the activation and maturation of dendritic cells in the lupus model. It is possible that salt does not promote expansion but rather changes the phenotypes and function of dendritic cells. We also observed that excessive salt stimulated dendritic cells to secrete proinflammatory cytokines and decreased the secretion of anti-inflammatory cytokines. Further investigation of the functions of dendritic cells that were pretreated with or without salt chloride and then transferred to induce lupus in recipient mice further supported our hypothesis. We found that the in vitro pretreatment of dendritic cells with salt chloride resulted in a more potent capacity to induce lupus, a finding that was similar to the in vivo observation in lupus mice given excessive dietary salt orally. These results are consistent with previous studies of the phenotype and function of dendritic cells that indicated that dendritic cells are essential in immune reactions and that dendritic cell dysfunction results in autoimmune diseases.^80,81^

Xiong and colleagues systemically studied the biological features of ALD-DNA-induced murine lupus.^51–55,82–88^ Interestingly, hypomethylated DNA as emerged in patients with SLE.^89,90^ ALD-DNA is extracted from Con-A-induced apoptotic splenocytes, and the methylation of ALD-DNA is lower than that of UALD-DNA (un-activated lymphocyte-derived DNA). Thus, hypomethylation may contribute to the immunogenicity of ALD-DNA, inducing SLE-like autoimmune disease in mice that are not susceptible to SLE.^91,92^ In addition, apoptotic B cells accelerate the onset of murine lupus in the preautoimmune (NZBxNZW)F1 strain.^93^ In our system, autoimmune responses are not initiated by apoptotic DC injection. Although uptake of apoptotic cells is generally tolerogenic,^94^ when apoptotic cells are not cleared and become necrotic, they then become immunogenic.^95^ However, this seems to not be a case in the current model. We found that anti-dsDNA levels were increased in BMDC-ALD-DNA-injected mice, while injected BMDCs were minimally apoptotic, distributed to various important organs and survived well. Moreover, we found that anti-dsDNA production occurred earlier than proteinuria and kidney pathology.^50^ Additionally, using wild-type mice, an HSD similarly enhanced fluid intake but did not cause anti-dsDNA production and proteinuria (data not shown). These results suggest that an HSD promotes lupus and renal pathology mainly through DC and immune cell activation but not via increased fluid intake. DC death is also less associated with inflammation and lupus-like findings in this model.

In addition, we also observed that excessive dietary salt promotes the activation and expansion of Tfh, B cell, plasma, and germinal center B cells during lupus development. It is possible that excessive salt promotes these cells directly or indirectly by enhancing antigen presentation capacity and cytokine production. We favor the indirect mechanism, since our study demonstrated that excessive salt does increase both the antigen presentation and proinflammatory cytokine production abilities of dendritic cells. These modified dendritic cells subsequently intensify the differentiation of different Th cell subsets, including Tfh cells. Consistent with this, OX40, which is a key protein that amplifies Tfh cell function,^96^ is expressed more highly on Tfh cells from lupus mice that received NaCl-treated dendritic cells. Conversely, CD62L expression was reduced, which is negatively related to the activation of Tfh cells.^97^ Given the importance of B cells in autoimmune diseases, we also studied the status of B cells, plasma cells, and germinal center B cells in lupus mice after receiving excessive dietary salt. As expected, the frequency of B cells, plasma cells, and germinal center B cells increased following high-salt administration. Since it is well established that germinal center B cells are crucial in the development of plasma cells and memory B cells,^98^ we focused on this particular cell population. An increase in germinal center B cells is highly relevant to the development of lupus, as we observed that an excessive salt diet significantly increased the levels of autoantibodies, including anti-dsDNA IgG and IgG2b, as well as IgG deposits in the kidney. We cannot exclude the possibility that high-salt intake affects the functional activity of CD4+ Foxp3+ Treg cells in our lupus model. In fact, a previous study demonstrated that an HSD did not affect Foxp3 expression but abolished the function of thymus-derived natural Treg cells.^31^ However, Treg cells consist of various populations, and high-salt intake has different roles in natural and induced Treg cells. The role of Treg cells in our lupus model deserves further study, and it will be interesting to learn whether an HSD changes Treg cell functions.

Mechanistically, we observed that STAT 1 and p38 MAPK were increased in dendritic cells after NaCl treatment at either the transcriptional or protein level. We found that the ratio of phospho-STAT1/total STAT1 showed no differences between blank BMDCs and NaCl-treated BMDCs, while the ratio of phospho-p38 MAPK/total p38 MAPK increased in NaCl-treated BMDCs compared with that of blank BMDCs, indicating that NaCl increased the expression of STAT1, as well as the expression and activation of p38 MAPK in BMDCs. P38 MAPK–STAT1 plays a significant role in dendritic cell maturation and function.^99,100^ We used p38 MAPK or STAT1 inhibitors to verify the role of these specific signaling molecules in the effects of high salt on dendritic cells. The activation or maturation marker, cytokine production, and antigen presentation ability of high-salt-treated dendritic cells diminished to normal levels when either the p38 MAPK or STAT1 inhibitor was added to the cultures. Moreover, p38 MAPK or STAT1 inhibitor treatment also abolished the enhanced effect of sodium chloride on the dendritic cell-initiated lupus model. Moreover, we treated STAT1 knockout BMDCs with NaCl and found that NaCl did not facilitate BMDC^STAT1−/−^-ALD-DNA-induced murine lupus and that NaCl did not exacerbate the activation and maturation of BMDCs in vitro. However, the mechanism by which NaCl acts on dendritic cells remains elusive and warrants independent study in the future.

As this observation was made in animal models, the role of HSD in patients with SLE or other autoimmune diseases merits further epidemiological study, as it has important clinical implications regarding diet and disease development. We therefore propose that normal- or low-salt intake be recommended to patients with SLE as an approach to reduce the incidence of flares and improve therapeutic effects and prognosis.

Taken together, we demonstrated that sodium chloride upregulates the immune ability of dendritic cells through the p38 MAPK and STAT1 signaling pathways and subsequently exacerbates murine SLE. This finding is significant in developing novel options for the prevention and treatment of patients with SLE and other dendritic cell-mediated diseases.

## Methods

### Mice

SLE-prone NZM2328 mice were originally obtained from the Jackson Laboratory and were housed and developed in the Stohl/Zheng lab at the University of Southern California and then in the Zheng lab at Ohio State University. C57BL/6 (female, 8–10 weeks) and Balb/c mice were obtained from Beijing Vital River Laboratory Animal Technology Co., Ltd. All mice were housed in the Center of Experimental Animals of Sun Yat-sen University or Ohio State University. STAT1 knockout mice were a gift from Professor Xinyuan Fu. The mice received normal chow and tap water (NSD group) or sodium-rich chow containing 4% NaCl (Guangdong Animal Experiment Center; catalog number 20190217) and tap water containing 1% NaCl as previously decribed.^79,101^ Five mice were used in each group in each experiment, and the experiments were repeated at least three times with similar results. The use of animals was approved by the Institutional Animal Care and Use Committee of Sun Yat-sen University and Ohio State University.

### Dendritic cell induction

Bone marrow cells were obtained from C57BL/6 mice and were separated and prepared as single-cell suspensions; erythrocytes were lysed with ACK buffer. The residual bone marrow cells were cultured in RPMI 1640 containing 10% heat-inactivated fetal bovine serum (HyClone), 100 IU/ml penicillin (Gibco), 1% sodium pyruvate (Corning), and 1% HEPES in the presence of 50 ng/ml rm-GM-CSF (Peprotech) and 2.5 ng/ml rm-IL-4 (Peprotech) to induce the differentiation of dendritic cells. After 6 days of incubation at 37 °C and 5% CO_2_, the dendritic cells were identified by using FACS staining with specific fluorescence-labeled CD11c antibody (Biolegend). The dendritic cells were incubated with ALD-DNA overnight with or without additional NaCl (20 mM, Sigma)^31,32,70,101^ and with or without the STAT1 inhibitor fludarabine (2 μg/ml, Selleck-21679-14-1) and p38 MAPK kinase inhibitor (5 μM, InvivoGen-tlrl-sb20). The harvested dendritic cells were collected for transfer to C57BL/6 mice or for flow cytometric measurement of activation and/or maturation markers.

### ALD-DNA preparation

As described previously by Xiong,^51^ splenocytes were stimulated with con-A (Sigma-Aldrich) for 6 days to reach an apoptotic status. Genomic DNA from apoptotic splenocytes was treated with S1 nuclease (Takara Bio) and proteinase K (Sigma-Aldrich) and then purified using DNeasy blood and tissue kits (Qiagen) according to the manufacturer’s instructions. The concentration of DNA was determined by a Nano-drop (Thermo Scientific). The final *A*_260_/*A*_280_ for the DNA preparations was >1.8. This preparation was termed activated syngeneic lymphocyte-derived DNA (ALD-DNA).

### Murine lupus model induction

As previously described,^50^ the lupus model was induced by transferring BMDCs that had been incubated with 50 μg/ml ALD-DNA into C57BL/6 mice; a total of 0.5 × 10^6^ dendritic cells were intravenously administered to each recipient mouse. Several separate mouse experiments were performed: (1) BMDC-ALD-DNA was injected into mice in the NSD or HSD group. (2) BMDC-ALD-DNA was incubated with or without NaCl and injected into mice, and the ratio of dendritic cells from the control or NaCl pretreatment group was similar (Supplementary Fig. 6b). (3) BMDC-ALD-DNA was incubated with or without NaCl and with or without p38 MAPK inhibitor or STAT1 inhibitor and then injected into mice. (4) STAT1 knockout mouse-derived BMDC-ALD-DNA (BMDC^STAT1−/−^-ALD-DNA) was incubated with or without NaCl and injected into mice. Proteinuria was determined with semiquantitative Albustix paper (Gaoerbao).

### Flow cytometric analysis

The cells were resuspended in ice-cold PBS containing 1% FBS and were stained with anti-CD3, anti-CD4, anti-CD8, anti-B220, anti-CD38, anti-CD138, anti-PD-1, anti-CXCR5, anti-CD62L, anti-OX40, anti-IgD, anti-CD95, anti-GL7, anti-CD11c, anti-CD80, anti-CD86, anti-MHC II, anti-CD40, anti-CD69, anti-CD11b, anti-Gr-1, and anti-F4/80 antibodies (Biolegend). Intracellular staining of IFN-γ, TNF-α, IL-17a, Helios, and Foxp3 was performed after 4 h of stimulation with PMA and ionomycin following a standard protocol (BD Biosciences). The results were obtained on a BD FACS Fortessa flow cytometer and were analyzed using FlowJo software.

### DC-T cell antigen presentation experiments

BMDCs from C57BL/6 mice were pretreated with 10 ng/ml LPS and/or 20 mM NaCl and with or without p38 MAPK inhibitor or STAT1 inhibitor for 24 h and then collected for coculture with T cells. T cells were isolated from the spleens and lymph nodes of Balb/c mice and were labeled with CFSE using the CellTrace^TM^ CFSE cell proliferation kit (ThermoFisher Scientific). The labeled T cells were cocultured with dendritic cells, and T cell proliferation was determined after 72 h.

### BMDC tracking and apoptosis analysis

Cell-tracker CM-DIL (Invitrogen-C7000)-labeled BMDCs (5 × 10^6^ BMDCs per mouse) were transferred into normal C57BL/6 mice by tail vein injection and were imaged in vivo with an In Vivo Xtreme system (Bruker, Billerica, MA, USA). The mice were euthanized at day 7 and day 14 after cell transfer, and the organs were collected and imaged by an In Vivo Xtreme system. On days 7 and 14, the spleens were harvested, fixed in 100% acetone and 1% paraformaldehyde, and stained with TUNEL (green) and BMDC-DIL (red); TUNEL staining represents apoptosis and was observed with a fluorescence microscope (ZEISS, Germany).

### BMDC-ALD-DNA activation analysis in recipient mice

Dendritic cells were derived from bone marrow from CD45.1+ mice, incubated with ALD-DNA, and transferred to CD45.2+ recipient mice. 7 and 14 days after DC transfer, the experiments were ended, the spleens were harvested, and the activation markers (CD80, CD86, and MHC II) on donor BMDCs (CD45.1+ CD11C+) and recipient dendritic cells (CD45.1-CD11C+) were analyzed.

### Cytokine analysis

Cell culture supernatants were collected from cell culture plates at the indicated time points, and sera were collected from the different groups of lupus mice. IFN-γ, TNF, IL-17a, IL-2, IL-4, IL-6, and IL-10 concentrations were measured using a CBA kit (BD Biosciences) or ELISA kit (Life Invitrogen).

### Immunohistochemical kidney analysis

Experiments were ended at week 18 after dendritic cell injection, and the kidneys were removed, fixed in formalin, embedded in paraffin, sectioned, and stained with hematoxylin and eosin, Masson’s trichrome, PAS, and PASM. H&E scoring represents the pathological lesions of nephritis and was graded on a semiquantitative scale ranging from 0 to 4: 0 = normal; 1 = mild hyperplasia of the glomerular mesangium; 2 = moderate level hyperplasia in the mesangium; 3 = glomerular lobular formation and thickened basement membrane; and 4 = glomerular crescent formation, sclerosis, tubular atrophy, and casts. Masson scoring represents interstitial fibrosis and was assessed in three randomly chosen fields at ×400 magnification from five mice per group and was scored by the following criteria: 1, <25% area of damage; 2, 25 –50% area of damage; 3, 50–75% area of damage; and 4, >75% area of damage. PAS scoring represents glomerular damage and was assessed in three randomly chosen fields at ×400 magnification from five mice per group and was scored as follows: 1, <25%; 2, 25–50%; 3, 50–75%; 4, >75%; and 5, completely sclerotic glomeruli.

### Immunofluorescence kidney analysis

Frozen sections were fixed in 100% acetone and 1% paraformaldehyde, stained with rabbit anti-mouse IgG (Abcam) and rabbit anti-mouse C3 (Abcam) and then stained with goat anti-rabbit IgG (Abcam). The sections were observed with a fluorescence microscope (ZEISS, Germany), and the MFI of glomeruli in different groups was calculated using ImageJ software (National Institutes of Health, USA).

### ELISA

The sera were collected by retro-orbital venous plexus collection at weeks 0, 4, 8, 12 and 16 after the BMDC-ALD-DNA was transferred to the recipient mice. Anti-dsDNA antibodies were detected by ELISA, recognizing that this technique has the potential for some nonspecific binding.^102^ Ninety-six-well plates were coated with 200 μg/ml salmon DNA (Sigma) overnight at 37 °C. After washing four times with PBS containing 0.05% Tween-20 (Sigma) and then blocking with 10% fetal bovine serum (HyClone) in PBS for 1 h, the serum was diluted 100-fold in 1% BSA. The samples were added and incubated for 2 h at room temperature, washed once and primed with horseradish peroxidase (HRP)-conjugated goat anti-mouse IgG, IgG1, IgG2a, or IgG2b antibody. After washing seven times, the color development was primed with TMB (Sigma-Aldrich), the reaction was stopped by 0.5 M H_2_SO_4_, and the absorbance at 450 nm (OD450) was measured in a microplate reader (Bio-Rad).

### Western blotting

Dendritic cells were collected and lysed with lysing buffer (Sigma). Protein extracts were separated on 10% polyacrylamide-SDS gels and electroblotted onto nitrocellulose membranes (Gene script). After blocking with 5% nonfat dry milk in TBS, the membranes were incubated with antibodies against STAT1, P38 MAPK, phosphorylated STAT1, and phosphorylated p38 MAPK (Cell Signaling Technology), followed by incubation with HRP-conjugated secondary antibody (Cell Signaling Technology). The blots were normalized to GAPDH.

### RT-PCR

Total RNA was extracted from dendritic cells with the RNeasy mini kit (Omega, R6834-2), and cDNA was generated by using a PrimeScriptTM RT-PCR kit (TAKARA). STAT1, STAT3, STAT4, STAT5, Caspase1, Caspase3, Caspase8, Caspase9, JAK1, JAK2, IRF5, IL-6, IL-23a, IL-1a, TLR4, TLR7, and TLR9 mRNA expression was quantified by using a SYBP ^○^R Premix Ex TaqTM II Kit (TAKARA). The samples were analyzed in triplicate, and the relative expression of the target genes was determined by normalizing the expression of each target gene to β-Actin by using the 2−ΔΔCt method.

### Statistical analysis

For comparison of treatment groups, we performed paired *t*-tests or the nonparametric Mann–Whitney test as indicated. All statistical analyses were performed using GraphPad Prism software. A *p* value <0.05 was considered statistically significant. Values represent the means ± SEM of three independent experiments (**p < 0.005, ***p < 0.0005, and ****p < 0.0001).

## Supplementary information

SUPPLEMENTAL MATERIAL

## Data Availability

The datasets generated and/or analyzed during the current study are available from the corresponding author upon reasonable request.

## References

[1] Chang NH (2008). Expanded population of activated antigen-engaged cells within the naive B cell compartment of patients with systemic lupus erythematosus. J. Immunol..

[2] Borchers AT (2012). Lupus nephritis: a critical review. Autoimmun. Rev..

[3] Rovin BH, Parikh SV (2014). Lupus nephritis: the evolving role of novel therapeutics. Am. J. Kidney Dis..

[4] D’Cruz D, Khamashta M, Hughes G (2007). Systemic lupus erythematosus. Lancet.

[5] Mu Q, Zhang H, Luo XM (2015). SLE: another autoimmune disorder influenced by microbes and diet?. Front. Immunol..

[6] Bakshi J, Segura BT, Wincup C, Rahman A (2018). Unmet needs in the pathogenesis and treatment of systemic lupus erythematosus. Clin. Rev. Allergy Immunol..

[7] Urbonaviciute V, Luo H, Sjöwall C, Bengtsson A, Holmdahl R (2019). Low production of reactive oxygen species drives systemic lupus erythematosus. Trends Mol. Med..

[8] Long H (2016). The critical role of epigenetics in systemic lupus erythematosus and autoimmunity. J. Autoimmun..

[9] Takasaki S (2007). Role of pathogenic auto-antibody production by Toll-like receptor 9 of B cells in active systemic lupus erythematosus. Rheumatology (Oxford).

[10] Karrar S, Cunninghame Graham DS (2018). Abnormal B cell development in systemic lupus erythematosus: what the genetics tell us. Arthritis Rheumatol..

[11] Morimoto S (2007). Expression of B-cell activating factor of the tumour necrosis factor family (BAFF) in T cells in active systemic lupus erythematosus: the role of BAFF in T cell-dependent B cell pathogenic autoantibody production. Rheumatology (Oxford).

[12] Yang J, Yang X, Zou H, Chu Y, Li M (2011). Recovery of the immune balance between Th17 and regulatory T cells as a treatment for systemic lupus erythematosus.. Rheumatology (Oxford).

[13] Mangini AJ, Lafyatis R, Van Seventer JM (2007). Type I interferons inhibition of inflammatory T helper cell responses in systemic lupus erythematosus. Ann. N. Y Acad. Sci. USA.

[14] Funauchi M, Ikoma S, Enomoto H, Horiuchi A (1998). Decreased Th1-like and increased Th2-like cells in systemic lupus erythematosus. Scand. J. Rheumatol..

[15] Guillaume MP, Hermanus N, Demulder A, Servais G, Karmali R (2003). Specific autoantibodies of SLE, such as anti-Ku, anti-ribosome Po and anti-membrane DNA autoantibodies, in a case of human African trypanosomiasis. Rheumatology (Oxford).

[16] Sturfelt G, Truedsson L (2005). Complement and its breakdown products in SLE. Rheumatology (Oxford).

[17] Fernández de Larrinoa IR-F (2015). What is new in systemic lupus erythematosus. Reumatol. Clin..

[18] Brilland, B., et al. Platelets and IgE: shaping the innate immune response in systemic lupus erythematosus. *Clin. Rev. Allergy Immunol*. 1–19 (2019). 10.1007/s12016-019-08744-x 31254159

[19] Suarez-Fueyo A, Bradley SJ, Tsokos GC (2016). T cells in systemic lupus erythematosus. Curr. Opin. Immunol..

[20] Yaniv G (2015). A volcanic explosion of autoantibodies in systemic lupus erythematosus: a diversity of 180 different antibodies found in SLE patients. Autoimmun. Rev..

[21] Leffler J, Bengtsson AA, Blom AM (2014). The complement system in systemic lupus erythematosus: an update. Ann. Rheum. Dis..

[22] Mageau A (2019). The burden of chronic kidney disease in systemic lupus erythematosus: a nationwide epidemiologic study. Autoimmun. Rev..

[23] Ichinose K (2017). Unmet needs in systemic lupus erythematosus. Nihon Rinsho Men’eki Gakkai kaishi=Jpn. J. Clin. Immunol..

[24] Murphy G, Isenberg DA (2019). New therapies for systemic lupus erythematosus—past imperfect, future tense. Nat. Rev. Rheumatol..

[25] Durcan L, O’Dwyer T, Petri M (2019). Management strategies and future directions for systemic lupus erythematosus in adults. Lancet.

[26] Chasset F, Francès C (2019). Current concepts and future approaches in the treatment of cutaneous lupus erythematosus: a comprehensive review. Drugs.

[27] Aytan J, Bukhari MA (2016). Use of biologics in SLE: a review of the evidence from a clinical perspective. Rheumatology (Oxford).

[28] Jörg S (2016). High salt drives Th17 responses in experimental autoimmune encephalomyelitis without impacting myeloid dendritic cells. Exp. Neurol..

[29] Paling D (2013). Sodium accumulation is associated with disability and a progressive course in multiple sclerosis. Brain.

[30] Haase S (2018). Sodium chloride triggers Th17 mediated autoimmunity. J. Neuroimmunol..

[31] Luo Y (2019). Negligible effect of sodium chloride on the development and function of TGF-β-induced CD4+ Foxp3+ regulatory T cells. Cell Rep..

[32] Hucke S (2016). Sodium chloride promotes pro-inflammatory macrophage polarization thereby aggravating CNS autoimmunity. J. Autoimmun..

[33] Wu H (2016). High salt promotes autoimmunity by TET2-induced DNA demethylation and driving the differentiation of Tfh cells. Sci. Rep..

[34] Simpson N (2010). Expansion of circulating T cells resembling follicular helper T cells is a fixed phenotype that identifies a subset of severe systemic lupus erythematosus. Arthritis Rheum..

[35] Yu D, Vinuesa CG (2010). Multiple checkpoints keep follicular helper T cells under control to prevent autoimmunity. Cell Mol. Immunol..

[36] Chen M (2012). The development and function of follicular helper T cells in immune responses. Cell. Mol. Immunol..

[37] Chen M (2014). The function of BAFF on T helper cells in autoimmunity. Cytokine Growth Factor Rev..

[38] Chen M (2018). Advances in T follicular helper and T follicular regulatory cells in transplantation immunity. Transpl. Rev. (Orlando).

[39] Said A, Weindl G (2015). Regulation of dendritic cell function in inflammation. J. Immunol. Res.

[40] Lan Q (2012). Polyclonal CD4+Foxp3+ Treg cells induce TGFβ-dependent tolerogenic dendritic cells that suppress the murine lupus-like syndrome. J. Mol. Cell Biol..

[41] Zheng SG (2006). Transfer of regulatory T cells generated ex vivo modifies graft rejection through induction of tolerogenic CD4+CD25+ cells in the recipient. Int. Immunol..

[42] Talay O, Shen CH, Chen L, Chen J (2009). B7-H1 (PD-L1) on T cells is required for T-cell-mediated conditioning of dendritic cell maturation. Proc. Natl. Acad. Sci. USA.

[43] RM S (2007). Dendritic cells: understanding immunogenicity. Eur. J. Immunol..

[44] Banchereau J, RM S (1998). Dendritic cells and the control of immunity. Nature.

[45] Morel PA (2013). Dendritic cell subsets in type 1 diabetes: friend or foe?. Front. Immunol..

[46] Bailey-Bucktrout SL (2008). Cutting edge central nervous system plasmacytoid dendritic cells regulate the severity of relapsing experimental autoimmune encephalomyelitis. J. Immunol..

[47] Mbongue J, Nicholas D, Firek A, Langridge W (2014). The role of dendritic cells in tissue-specific autoimmunity. J. Immunol. Res..

[48] Son M, Kim SJ, Diamond B (2016). SLE-associated risk factors affect DC function. Immunol. Rev..

[49] Sozzani S, Del Prete A, Bosisio D (2017). Dendritic cell recruitment and activation in autoimmunity. J. Autoimmun..

[50] Xiao Z (2017). Immunosuppressive effect of B7-H4 pathway in a murine systemic lupus erythematosus model. Front. Immunol..

[51] Qiao B (2005). Induction of systemic lupus erythematosus-like syndrome in syngeneic mice by immunization with activated lymphocyte-derived DNA. Rheumatology (Oxford).

[52] Zhang W, Xu W, Xiong S (2010). Blockade of Notch1 signaling alleviates murine lupus via blunting macrophage activation and M2b polarization. J. Immunol..

[53] Lu M (2015). HMGB1 promotes systemic lupus erythematosus by enhancing macrophage inflammatory response. J. Immunol. Res..

[54] Zhang W, Xu W, Xiong S (2011). Macrophage differentiation and polarization via phosphatidylinositol 3-kinase/Akt-ERK signaling pathway conferred by serum amyloid P component. J. Immunol..

[55] Wen Z, Xu L, Xu W, Xiong S (2012). Production of anti-double-stranded DNA antibodies in activated lymphocyte derived DNA induced lupus model was dependent on CD4+ T cells. Lupus.

[56] Zheng X (2019). Dendritic cell-associated B7-H3 suppresses the production of autoantibodies and renal inflammation in a mouse model of systemic lupus erythematosus. Cell Death Dis..

[57] Waters ST (2001). NZM2328: a new mouse model of systemic lupus erythematosus with unique genetic susceptibility loci. Clin. Immunol..

[58] Agrawal H (2009). Deficiency of type I IFN receptor in lupus-prone New Zealand mixed 2328 mice decreases dendritic cell numbers and activation and protects from disease. J. Immunol..

[59] Jacob N (2009). Accelerated pathological and clinical nephritis in systemic lupus erythematosus-prone New Zealand Mixed 2328 mice doubly deficient in TNF receptor 1 and TNF receptor 2 via a Th17-associated pathway. J. Immunol..

[60] McDole JR (2012). Goblet cells deliver luminal antigen to CD103+ dendritic cells in the small intestine. Nature.

[61] Domeier PP (2016). IFN-gamma receptor and STAT1 signaling in B cells are central to spontaneous germinal center formation and autoimmunity. J. Exp. Med..

[62] Awe O (2015). PU.1 expression in T follicular helper cells limits CD40L-dependent germinal center B cell development. J. Immunol..

[63] Wu H (2015). An inhibitory role for the transcription factor Stat3 in controlling IL-4 and Bcl6 expression in follicular helper T cells. J. Immunol..

[64] Hams E (2011). Blockade of B7-H1 (programmed death ligand 1) enhances humoral immunity by positively regulating the generation of T follicular helper cells. J. Immunol..

[65] Miyara M (2005). Global natural regulatory T cell depletion in active systemic lupus erythematosus. J. Immunol..

[66] Ma J (2010). The imbalance between regulatory and IL-17-secreting CD4+ T cells in lupus patients. Clin. Rheumatol..

[67] Zhao SS (2008). Expression of CD4+ CD25+ CD127(low/-) T cells in patients with systemic lupus erythematosus. Zhonghua Yi Xue Za Zhi..

[68] Zhong H (2018). TGF-beta-induced CD8(+)CD103(+) regulatory T cells show potent therapeutic effect on chronic graft-versus-host disease lupus by suppressing B cells. Front. Immunol..

[69] Zheng SG (2004). CD4+ and CD8+ regulatory T cells generated ex vivo with IL-2 and TGF-beta suppress a stimulatory graft-versus-host disease with a lupus-like syndrome. J. Immunol..

[70] Hernandez AL (2015). Sodium chloride inhibits the suppressive function of FOXP3+ regulatory T cells. J. Clin. Invest.

[71] Weigel BJ (2002). Comparative analysis of murine marrow-derived dendritic cells generated by Flt3L or GM-CSF/IL-4 and matured with immune stimulatory agents on the in vivo induction of antileukemia responses. Blood.

[72] Inaba K, Young JW, Steinman RM (1987). Direct activation of CD8+ cytotoxic T lymphocytes by dendritic cells. J. Exp. Med..

[73] Luo M (2017). Simultaneous enhancement of cellular and humoral immunity by the high salt formulation of Al(OH)(3) adjuvant. Cell Res..

[74] Ramalingam R (2012). Dendritic cell-specific disruption of TGF-beta receptor II leads to altered regulatory T cell phenotype and spontaneous multiorgan autoimmunity. J. Immunol..

[75] Lin. X (2013). Advances in distinguishing natural from induced Foxp3(+) regulatory T cells. Int J. Clin. Exp. Pathol..

[76] Thornton AM (2010). Expression of Helios, an Ikaros transcription factor family member, differentiates thymic-derived from peripherally induced Foxp3+ T regulatory cells. J. Immunol..

[77] Weiqian C (2017). A protocol to develop T helper and Treg cells in vivo. Cell. Mol. Immunol..

[78] Goh KC, Haque SJ, Williams BR (1999). p38 MAP kinase is required for STAT1 serine phosphorylation and transcriptional activation induced by interferons. EMBO J..

[79] Kleinewietfeld M (2013). Sodium chloride drives autoimmune disease by the induction of pathogenic TH17 cells. Nature.

[80] Maddur MS (2010). Dendritic cells in autoimmune diseases. Open Arthritis J..

[81] Mackern-Oberti JP (2014). Targeting dendritic cell function during systemic autoimmunity to restore tolerance. Int. J. Mol. Sci..

[82] Cai Y, Zhang W, Xiong S (2013). Mannose-binding lectin blunts macrophage polarization and ameliorates lupus nephritis. PLoS One.

[83] Chen M (2011). Blockade of TLR9 signaling in B cells impaired anti-dsDNA antibody production in mice induced by activated syngenic lymphocyte-derived DNA immunization. Mol. Immunol..

[84] Chen X, Wen Z, Xu W, Xiong S (2013). Granulin exacerbates lupus nephritis via enhancing macrophage M2b polarization. PLoS One.

[85] Zhang W (2012). AIM2 facilitates the apoptotic DNA-induced systemic lupus erythematosus via arbitrating macrophage functional maturation. J. Clin. immunol..

[86] Wang Y (2006). Autoantibodies closely relate to the elevation level of in vivo hydrogen peroxide and tissue damage in systemic lupus erythematosus. DNA Cell Biol..

[87] Zhang W, Wu J, Qiao B, Xu W, Xiong S (2011). Amelioration of lupus nephritis by serum amyloid P component gene therapy with distinct mechanisms varied from different stage of the disease. PLoS One.

[88] Wen Z (2013). Interleukin-17 expression positively correlates with disease severity of lupus nephritis by increasing antidouble-stranded dna antibody production in a lupus model induced by activated lymphocyte derived DNA. PLOS One..

[89] Richardson B (1990). Evidence for impaired T cell DNA methylation in systemic lupus erythematosus and rheumatoid arthritis. Arthritis Rheum..

[90] Yung RL, Richardson BC (1994). Role of T cell DNA methylation in lupus syndromes. Lupus.

[91] Pisetsky DS, Grudier JP, Gilkeson GS (1990). A role for immunogenic DNA in the pathogenesis of systemic lupus erythematosus. Arthritis Rheum..

[92] Pisetsky DS (2019). The central role of nucleic acids in the pathogenesis of systemic lupus erythematosus. F1000Research.

[93] Trebeden-Negre H, Weill B, Fournier C, Batteux F (2003). B cell apoptosis accelerates the onset of murine lupus. Eur. J. Immunol..

[94] Kushwah R (2010). Uptake of apoptotic DC converts immature DC into tolerogenic DC that induce differentiation of Foxp3+ Treg. Eur. J. Immunol..

[95] Ma L (2005). Systemic autoimmune disease induced by dendritic cells that have captured necrotic but not apoptotic cells in susceptible mouse strains. Eur. J. Immunol..

[96] Tahiliani V (2017). OX40 cooperates with ICOS to amplify follicular Th cell development and germinal center reactions during infection. J. Immunol..

[97] Hamel KM (2010). B7-H1 expression on non-B and non-T cells promotes distinct effects on T- and B-cell responses in autoimmune arthritis. Eur. J. Immunol..

[98] Mesin L, Ersching J, Victora GD (2016). Germinal center B cell dynamics. Immunity.

[99] Dong L (2018). Mesenchymal stem cells inhibited dendritic cells via the regulation of STAT1 and STAT6 phosphorylation in experimental autoimmune uveitis. Curr. Mol. Med.

[100] Jackson SH (2004). Dendritic cell maturation requires STAT1 and is under feedback regulation by suppressors of cytokine signaling. J. Immunol..

[101] Zhang WC (2015). High salt primes a specific activation state of macrophages, M(Na). Cell Res..

[102] Isenberg D, Smeenk R (2001). Clinical laboratory assays for measuring anti-dsDNA antibodies. Where are we now?. Lupus.

