# Supplementary material for: High salt diet accelerates the progression of murine lupus through dendritic cells via the p38 MAPK and STAT1 signaling pathways
Source: Signal Transduct Target Ther. 2020 Apr 10;5:34. doi: 10.1038/s41392-020-0139-5 (PMC7145808; doi:10.1038/s41392-020-0139-5)
Supplement: Supplementary file 1 — SUPPLEMENTAL MATERIAL [file 41392_2020_139_MOESM1_ESM.pdf]

Supplementary Information
(This PDF contains supplementary figures S1-S12.)

High Salt Diet Accelerates the Progress of Murine Lupus through Dendritic Cells via P38MAPK and the STAT1 Signaling Pathway

Ze Xiu Xiao[1], Xiaojiang Hu[1], Ximei Zhang[1,2,3], Zhigang Chen[1], Julie Wang[2], Ke Jin[4], Feng Lin Cao[5], Baoqing Sun[6], Joseph A. Bellanti[7], Nancy Olsen[3] and Song Guo Zheng[2*]

[1]Department of Clinical Immunology, the Third Affiliated Hospital at the Sun Yat-sen University, Guangzhou, 510630, China
[2]Department of Internal Medicine, the Ohio State University College of Medicine and Wexner Medical Center, Columbus, OH, 43210, United States
[3]Department of Medicine, Penn State College of Medicine, Hershey, PA, 17033, United States
[4]Laboratory of Human Diseases and Immunotherapy, West China Hospital, Sichuan University Chengdu, China
[5]Department of Internal Medicine, the First Hospital of Harbin Medical University, Harbin, China
[6]Department of Allergy and Clinical Immunology, the First Affiliated Hospital of Guangzhou Medical University, Guangzhou, China.
[7]Department of Pediatrics and Microbiology-Immunology, Georgetown University Medical Center, Washington, DC, United States

*Correspondence to Song Guo Zheng, MD, PhD, Email: SongGuo.Zheng@osumc.edu

a

b

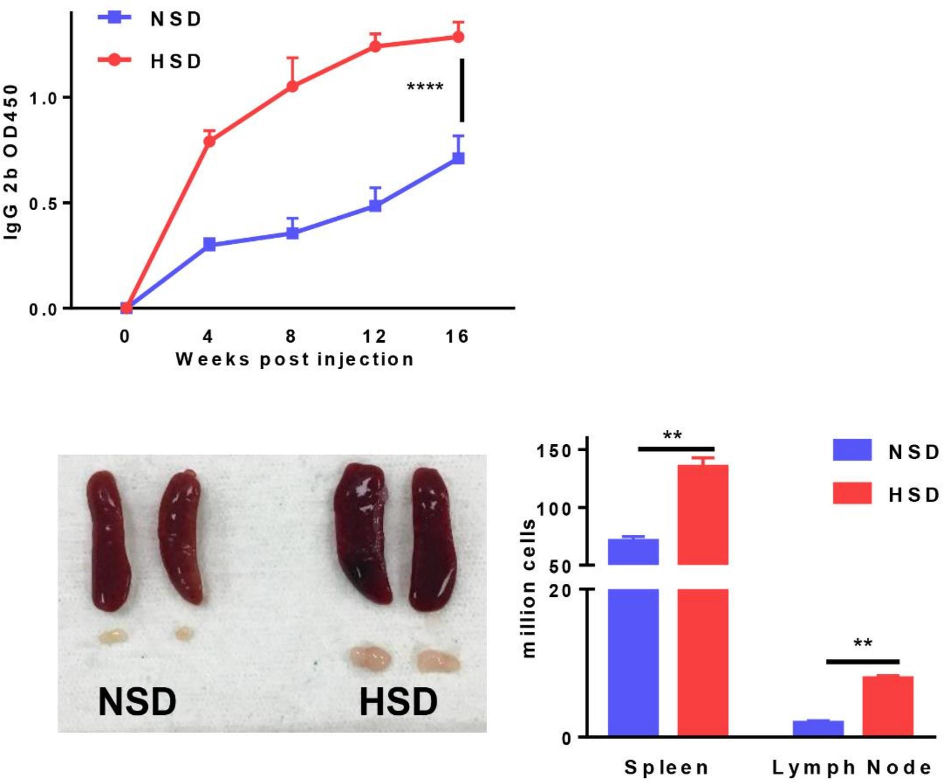

Figure S1. High salt diet enhanced the lupus syndromes of bone marrow cell-derived dendritic cells-ALD-DNA induced murine lupus model. Bone morrow derived dendritic cells incubated with ALD-DNA ($0.5x10^6$) were intravenously transferred to normal C57BL/6 mice, with normal salt diet (NSD) or high salt diet (HSD) in each individual experiment (n=5 in each individual experiment), experiments were ended at week 18. a. The semi-quantitative of IgG2b against ds-DNA in sera was measured with an ELISA between the HSD and NSD lupus group mice. b. The gross look (left) and the total cells number (right) of the spleens or lymph nodes from the NSD lupus mice *vs* HSD lupus mice. The results are displayed as the mean ± s.e.m. from three separate experiments. ns means no significance, **$p < 0.005$, ***$p < 0.0005$, **** < 0.0001 using non-parametric test Mann-Whitney tests.

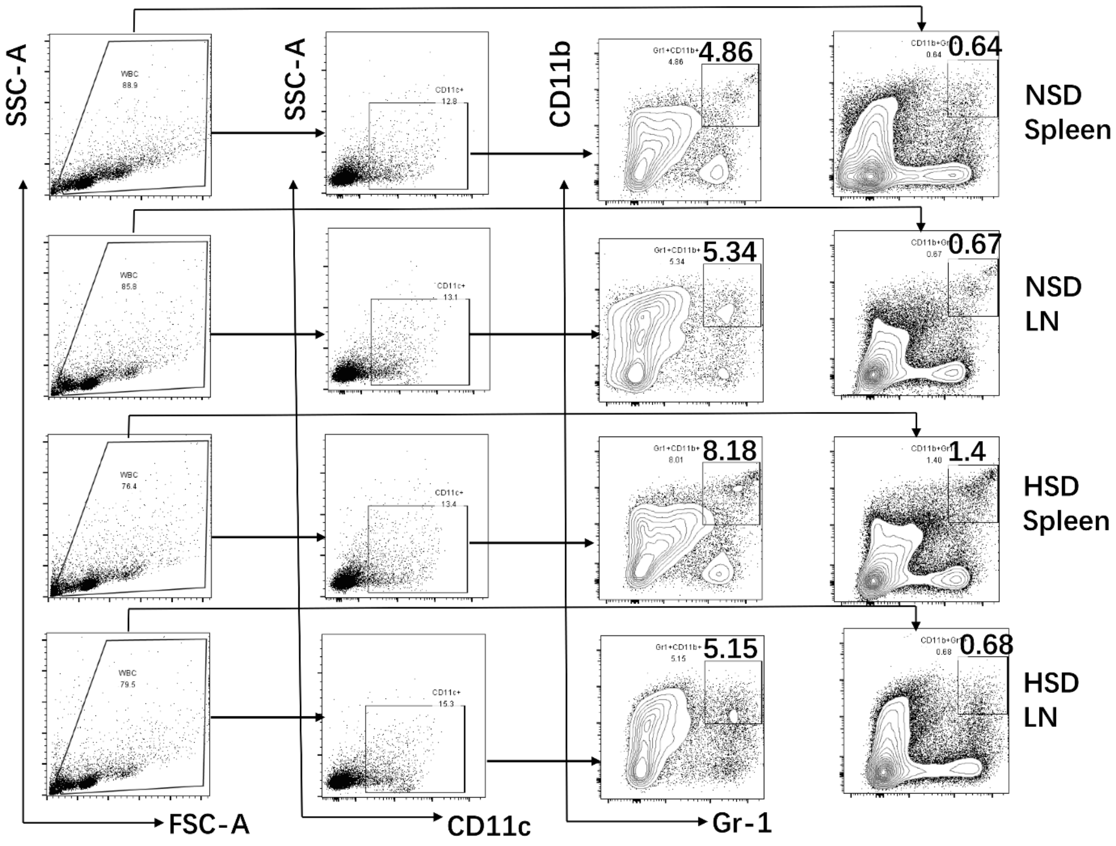

Figure S2. High salt diet did not influence the frequency of neutrophils from HSD lupus mice compared with NSD lupus mice. Bone morrow derived dendritic cells incubated with ALD-DNA ($0.5x10^6$) were intravenously transferred to normal C57BL/6 mice, with normal salt diet (NSD) or high salt diet (HSD). The frequency of neutrophils (CD11b+Gr-1+ marked or CD11c+ CD11b+Gr-1+ marked) was tested by flow cytometry on 4 weeks post the BMDC-ALD-DNA injection.

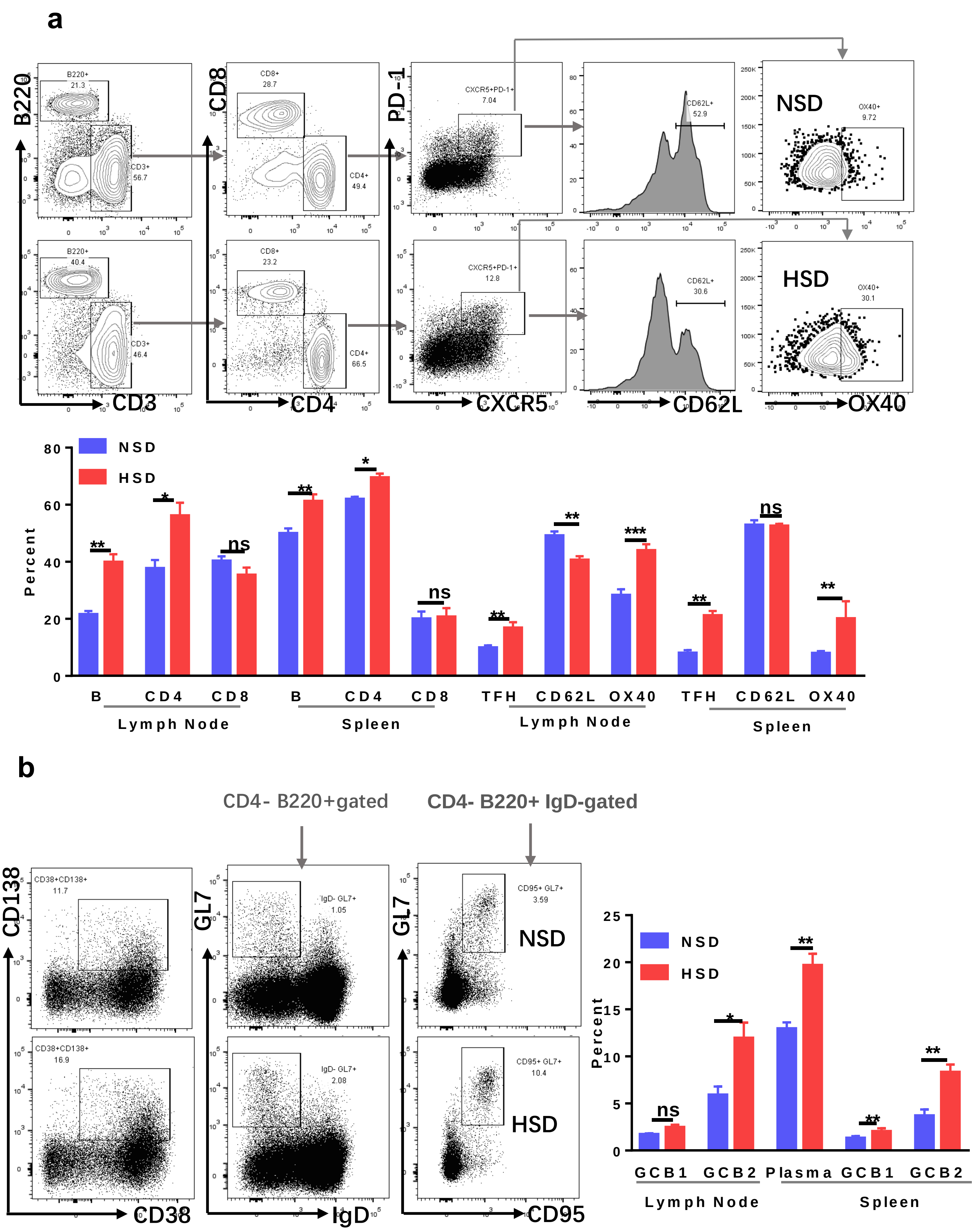
a
B220
CD3
CD8
CD4
PD-1
CXCR5
CD62L
OX40
NSD
HSD
B220+ 21.3
CD3+ 56.7
CD8+ 28.7
CD4+ 49.4
CXCR5+PD-1+ 7.04
CD62L+ 52.9
OX40+ 9.72
B220+ 40.4
CD3+ 46.4
CD8+ 23.2
CD4+ 66.5
CXCR5+PD-1+ 12.8
CD62L+ 30.6
OX40+ 30.1
Percent
B
CD4
CD8
TFH
CD62L
OX40
Lymph Node
Spleen
ns
b
CD4- B220+gated
CD4- B220+ IgD-gated
CD138
CD38
GL7
IgD
CD95
CD38+CD138+ 11.7
IgD- GL7+ 1.05
CD95+ GL7+ 3.59
CD38+CD138+ 16.9
IgD- GL7+ 2.08
CD95+ GL7+ 10.4
GCB1
GCB2
Plasma

c

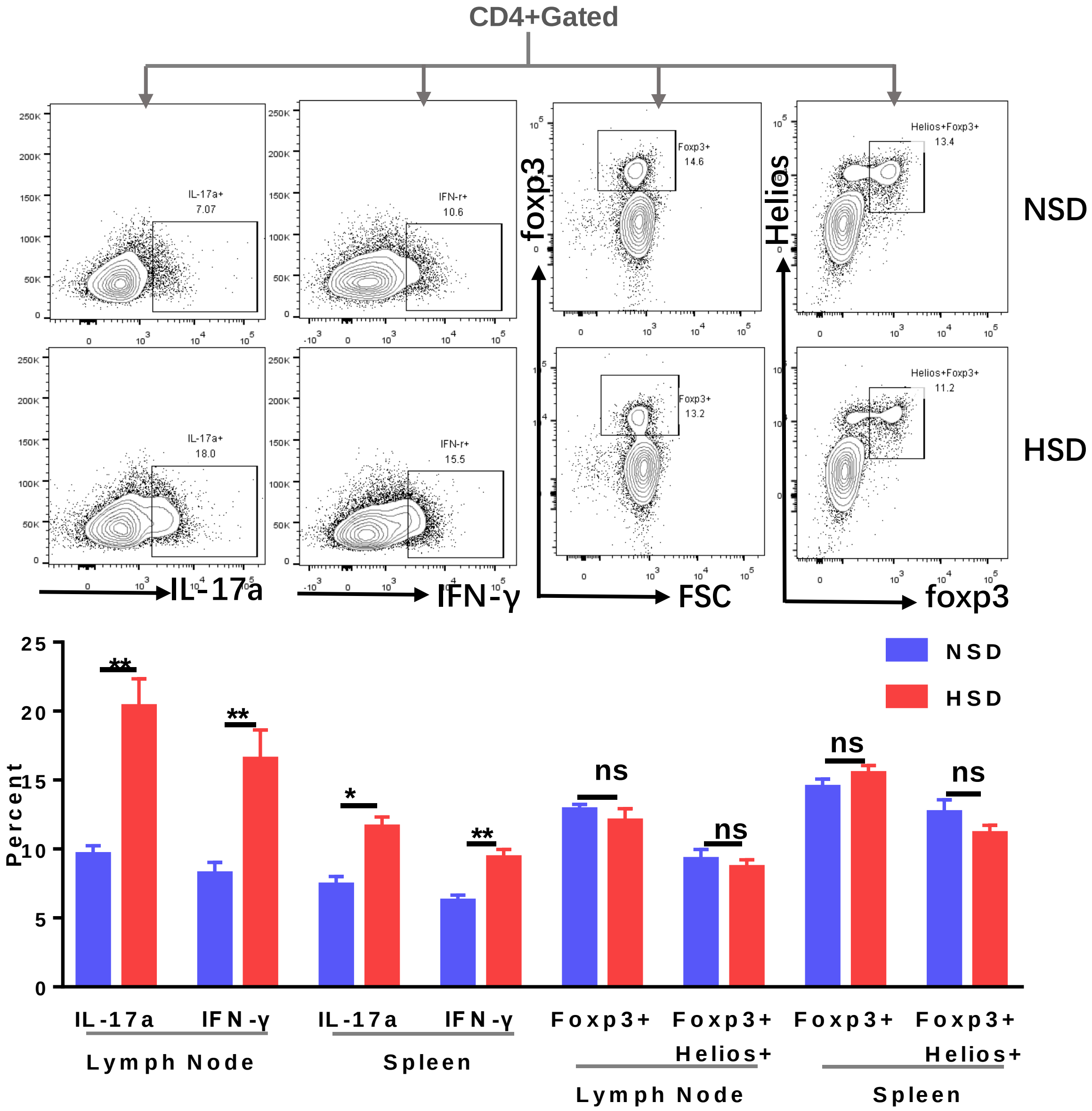

Figure S3. High salt diet increased the inflammatory cells in the bone marrow cell-derived dendritic cells-ALD-DNA induced murine lupus model. Bone morrow derived dendritic cells incubated with ALD-DNA ($0.5x10^6$) were intravenously transferred to normal C57BL/6 mice, with normal salt diet (NSD) or high salt diet (HSD) as described above (n=5 in each individual experiment). Flow cytometry was used to analyze the frequencies of B cells, CD4+ T cells and TFH cells (a), plasma cells and germinal center B cells (b), IL-17a+ T cells, IFN-γ+ T cells and regulatory T cells (nTregs and iTregs) (c) between the high salt diet and normal control salt diet mice. GCB cells were defined as (GCB1) CD4-B220+IgD-GL7+ [48] or (GCB2) CD4-B220+GL7+ CD95+ [49-51]. The results are displayed as the mean ± s.e.m. from three different experiments. ns means no significance, **p < 0.005, ***p < 0.0005, **** <

0.0001 using non-parametric test Mann-Whitney tests.

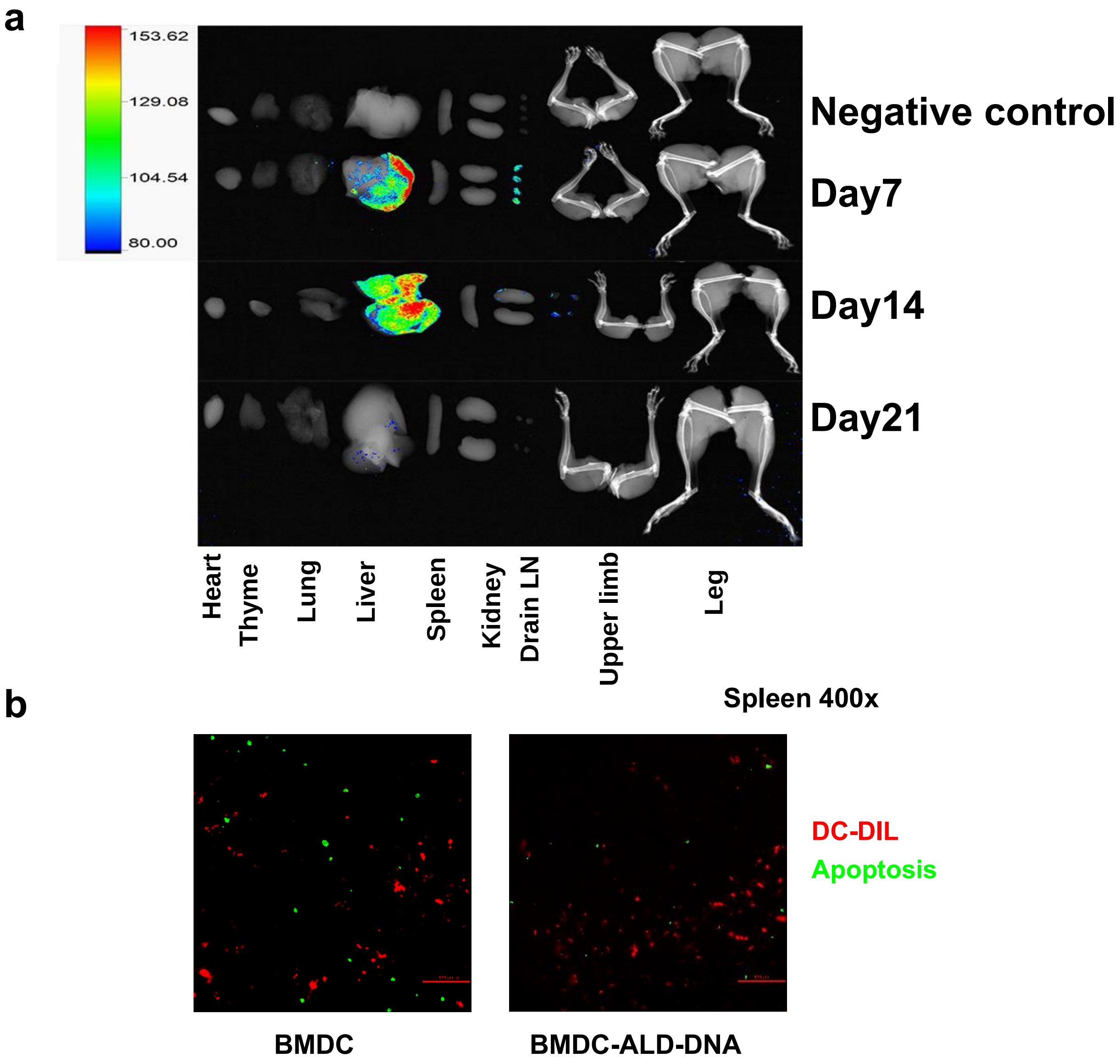

Figure S4. BMDCs survived well after adoptive transfer. a. Cell-tracker CM-DIL (Invitrogen-C7000) labeled BMDCs (5 x10^6) were transferred into normal C57BL/6 mice by tail intravenous injection and were *in vivo* imaged with xtreme *in-vivo* (Bruker, Germany). Mice were euthanatized at day 7, day 14, day 21 after cell transfer. Image demonstrated on xtreme was correlated with flow data, indicating BMDCs survived and participated in antigen-presenting for 2-3 weeks. b. Representative images demonstrating TUNEL (green) co-labeling with BMDC-DIL (red) in spleen after transplanted BMDC or BMDC-ALD-DNA at day 7, suggesting some apoptosis cells were not BMDCs, implicating the inflammation was not driven by apoptosis of BMDCs, rather than BMDC's antigen-presentation.

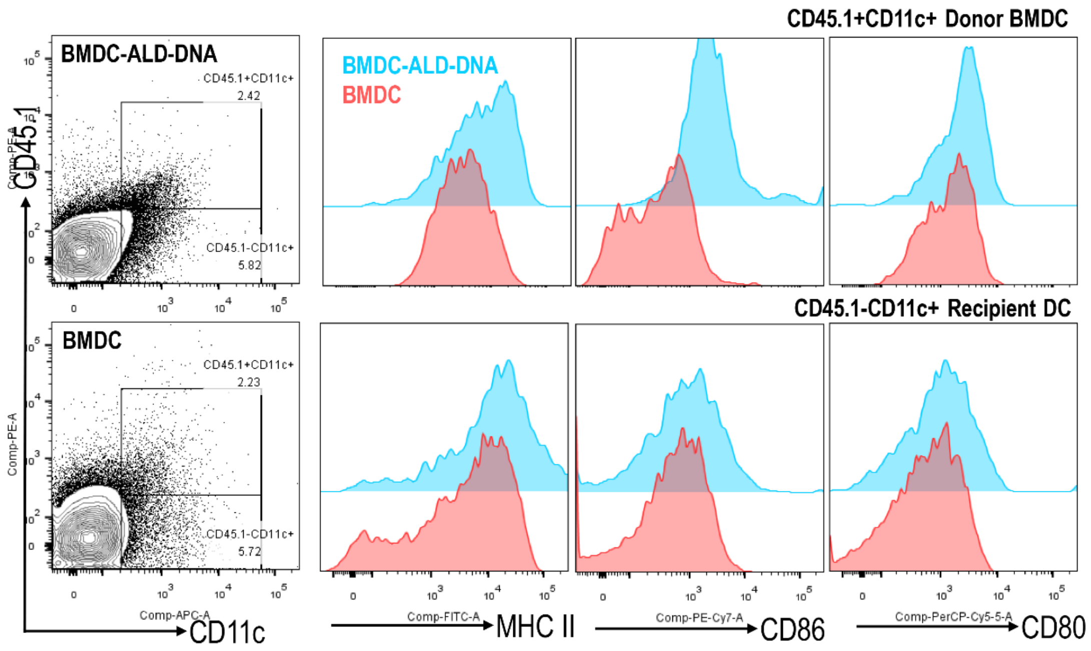

Figure S5. BMDCs-ALD-DNA activation analysis in recipient mice. Dendritic cells were derived from bone marrow in CD45.1+ mice, and then incubated with ALD-DNA and transferred to CD45.2+ recipient mice. 14 days after BMDC transfer, the experiments were ended and spleens were harvested and the activation maker (CD80, CD86, MHCII) on the donor DC (CD45.1+CD11C+) and recipient dendritic cells (CD45.1-CD11C+) were analyzed, respectively.

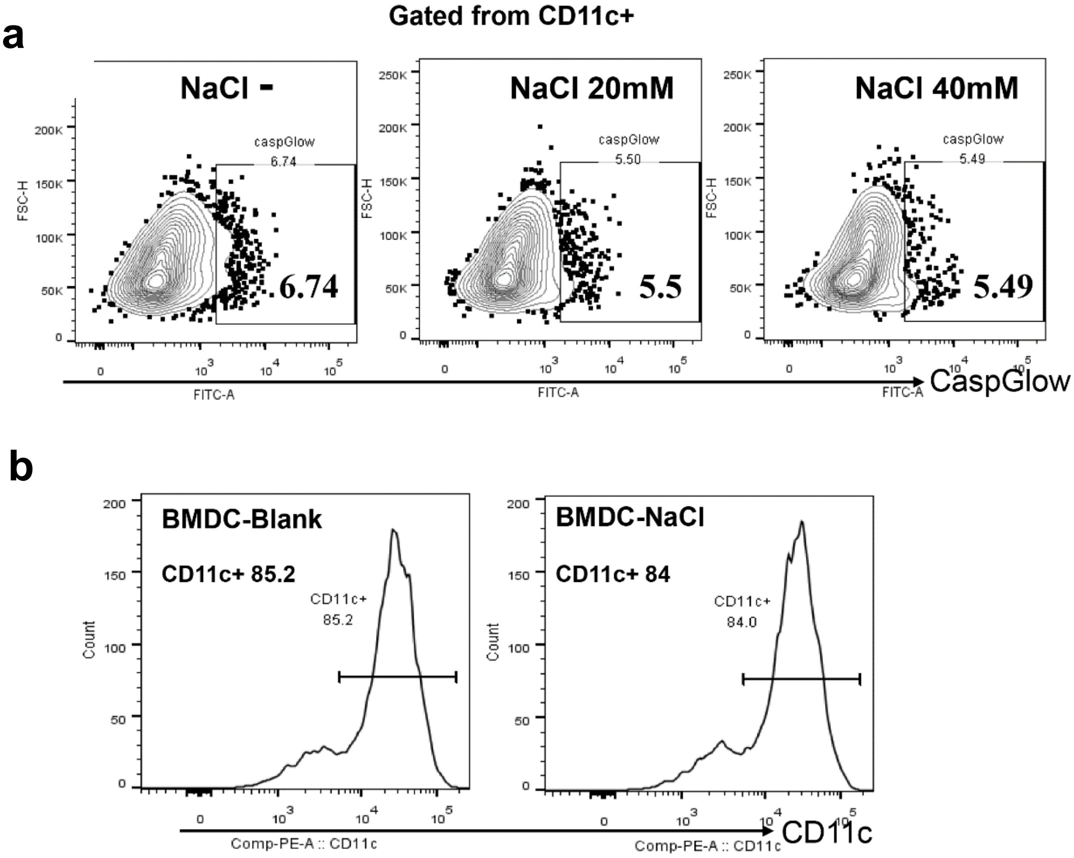

Figure S6. Bone morrow derived dendritic cells identification. a. Bone morrow derived dendritic cells were treated without or with additional NaCl 20 or 40 mM for 24 hours and using CaspGlow kit (Thermo Fisher) testified the death of the dendritic cells. b. Bone morrow derived dendritic cells were identified with Flow cytometry using CD11c fluorescent antibody before the cell transfer to the recipient mice after the incubation of ALD-DNA and pretreatment of NaCl. Data is a representative of 5 separate experiments.

a

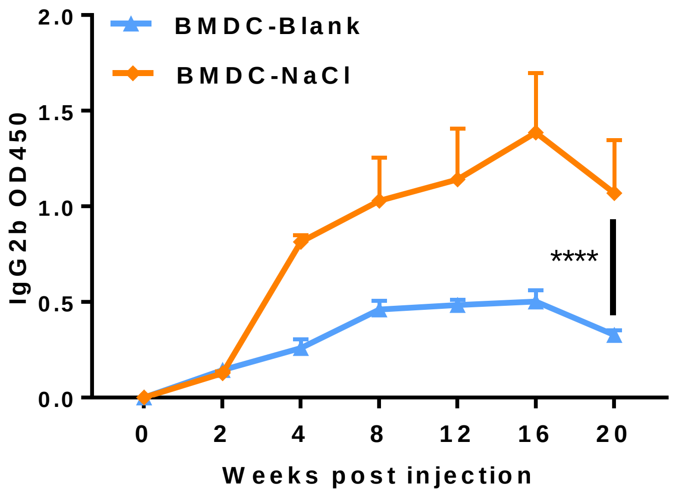

b

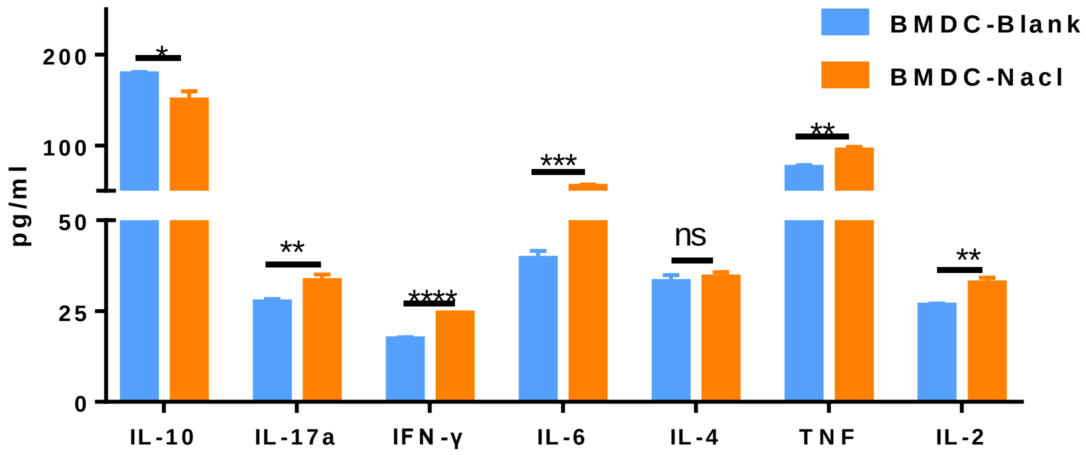

Figure S7. Bone marrow-derived dendritic cells pretreated with NaCl accelerated the syndromes of murine lupus model. Bone morrow derived dendritic cells incubated ALD-DNA were pretreated with or without NaCl (20mM) for 24 hours and these cells were harvested and widely washed, then transferred into normal C57BL/6 mice (n=5 in each individual experiment). a. ELISA experiment semi-quantitative of IgG2b against ds-DNA in sera from the NaCl-pretreated BMDCs-ALD-DNA induced lupus mice compared with control BMDCs-ALD-DNA induced lupus mice. b. CBA kit quantitative of cytokines in sera from the NaCl-pretreated BMDCs-ALD-DNA induced lupus mice compared with control BMDCs-ALD-DNA induced lupus mice. The results are displayed as the mean ± s.e.m. from three independent experiments. ns means no significance, **p < 0.005, ***p < 0.0005, **** < 0.0001 using non-parametric test Mann-Whitney tests.

a

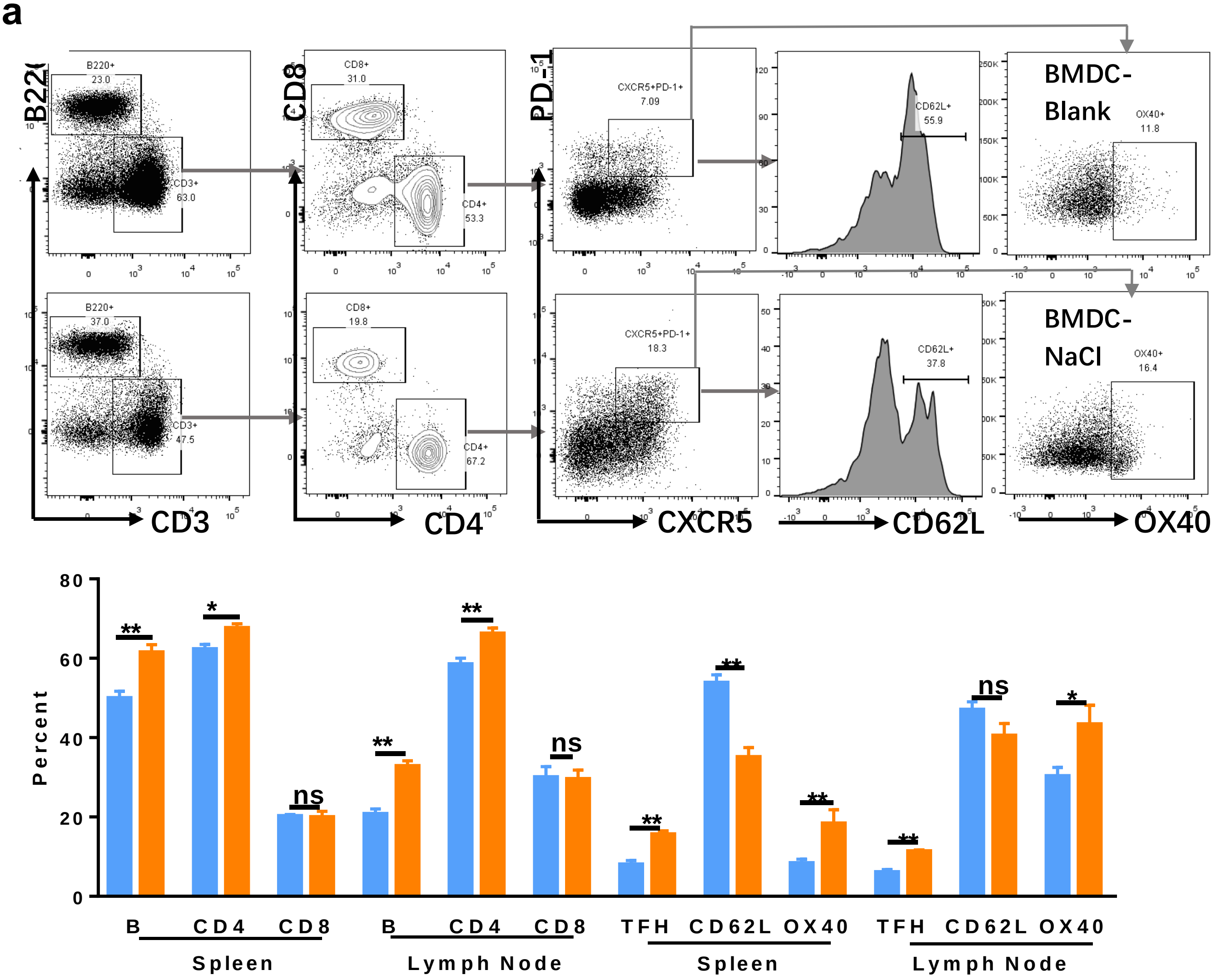
B220
CD3
CD8
CD4
PD-1
CXCR5
CD62L
OX40
BMDC-
Blank
BMDC-
NaCl
B220+
23.0
CD3+
63.0
B220+
37.0
CD3+
47.5
CD8+
31.0
CD4+
53.3
CD8+
19.8
CD4+
67.2
CXCR5+PD-1+
7.09
CXCR5+PD-1+
18.3
CD62L+
55.9
CD62L+
37.8
OX40+
11.8
OX40+
16.4
Percent
B
CD4
CD8
Spleen
Lymph Node
TFH
CD62L
OX40
ns
**
*

b

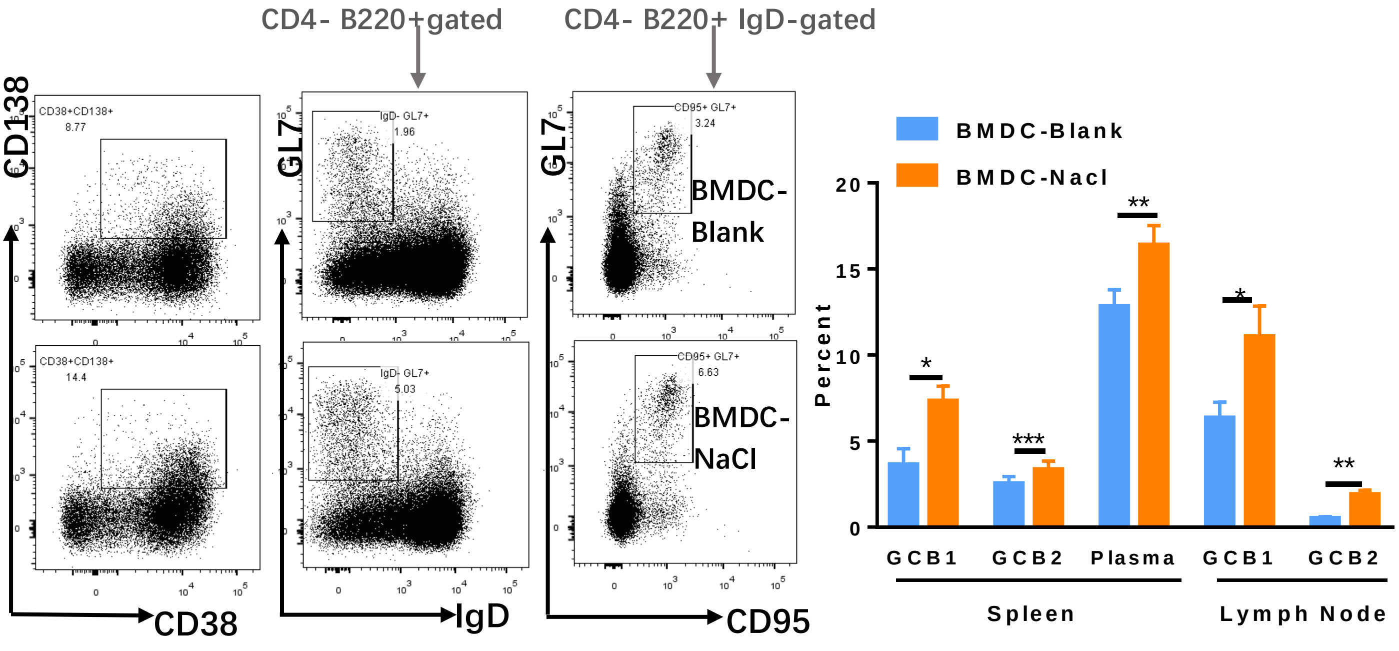
CD4- B220+gated
CD4- B220+ IgD-gated
CD138
CD38
GL7
IgD
CD95
CD38+CD138+
8.77
CD38+CD138+
14.4
IgD- GL7+
1.96
IgD- GL7+
5.03
CD95+ GL7+
3.24
CD95+ GL7+
6.63
BMDC-
Blank
BMDC-
NaCl
BMDC-Blank
BMDC-Nacl
Percent
GCB1
GCB2
Plasma
Spleen
Lymph Node
*
**
***

c

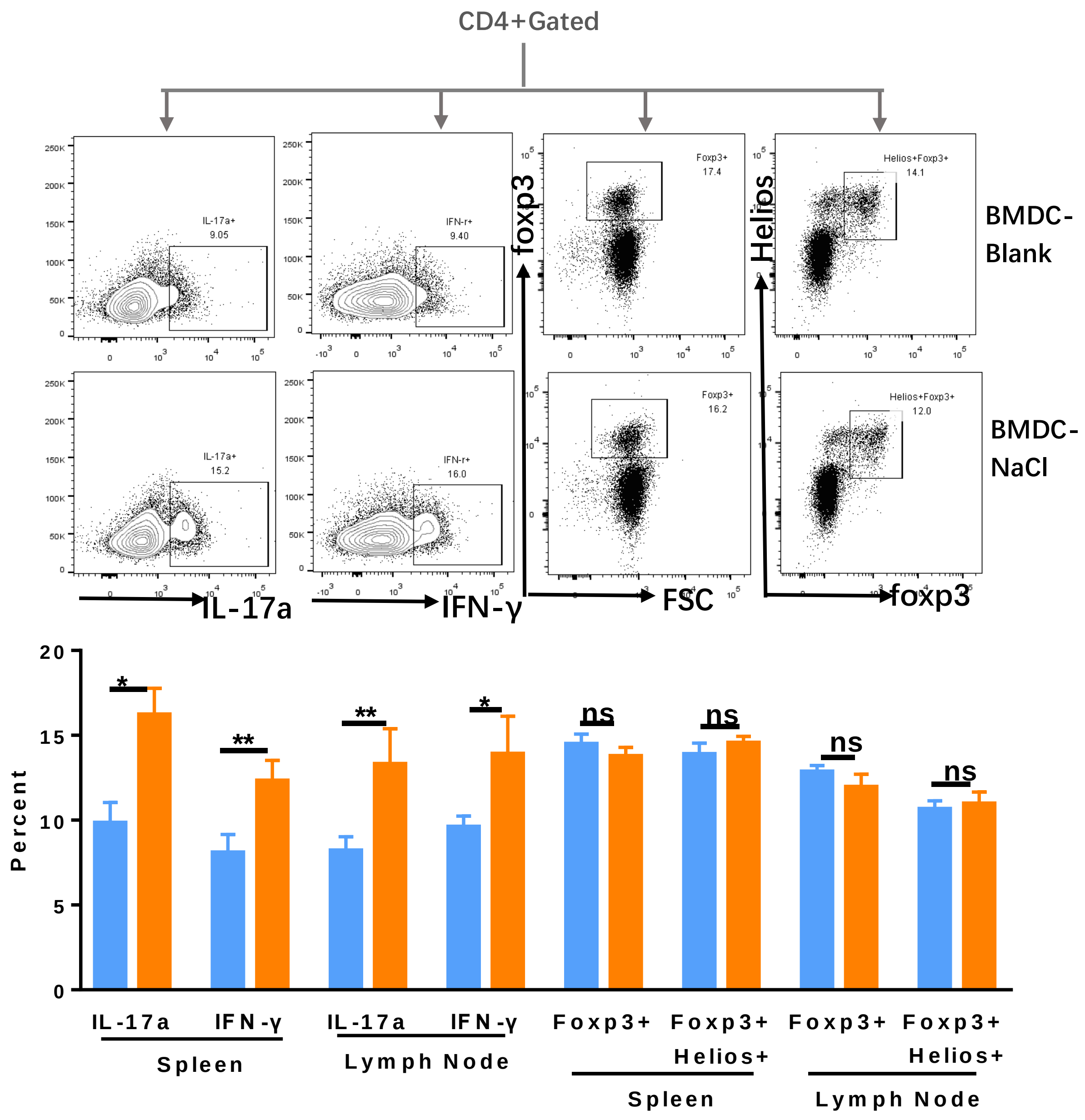

Figure S8. Increased the inflammatory cells from the BMDCs-ALD-DNA induced lupus mice compared with control BMDCs-ALD-DNA induced lupus. Bone morrow derived dendritic cells incubated ALD-DNA with or without treatment of NaCl(20mM) and then transferred to normal C57BL/6 mice (n=5 in each individual experiment). Flow cytometry analyzed B cells, CD4+ T cells and TFH cells (a), plasma cells and germinal center B cells (b), IL-17a+ T cells, IFN-γ+ T cells and regulatory T cells (nTregs and iTregs) (c) from the BMDCs-ALD-DNA induced lupus mice compared with control BMDCs-ALD-DNA induced lupus. GCB cells were defined as (GCB1) CD4-B220+IgD-GL7+ [48] or (GCB2) CD4-B220+GL7+ CD95+ [49-51]. The results are displayed as the mean ± s.e.m. from three different experiments. ns means no significance, **p < 0.005, ***p < 0.0005, **** < 0.0001 using non-parametric test Mann-Whitney tests.

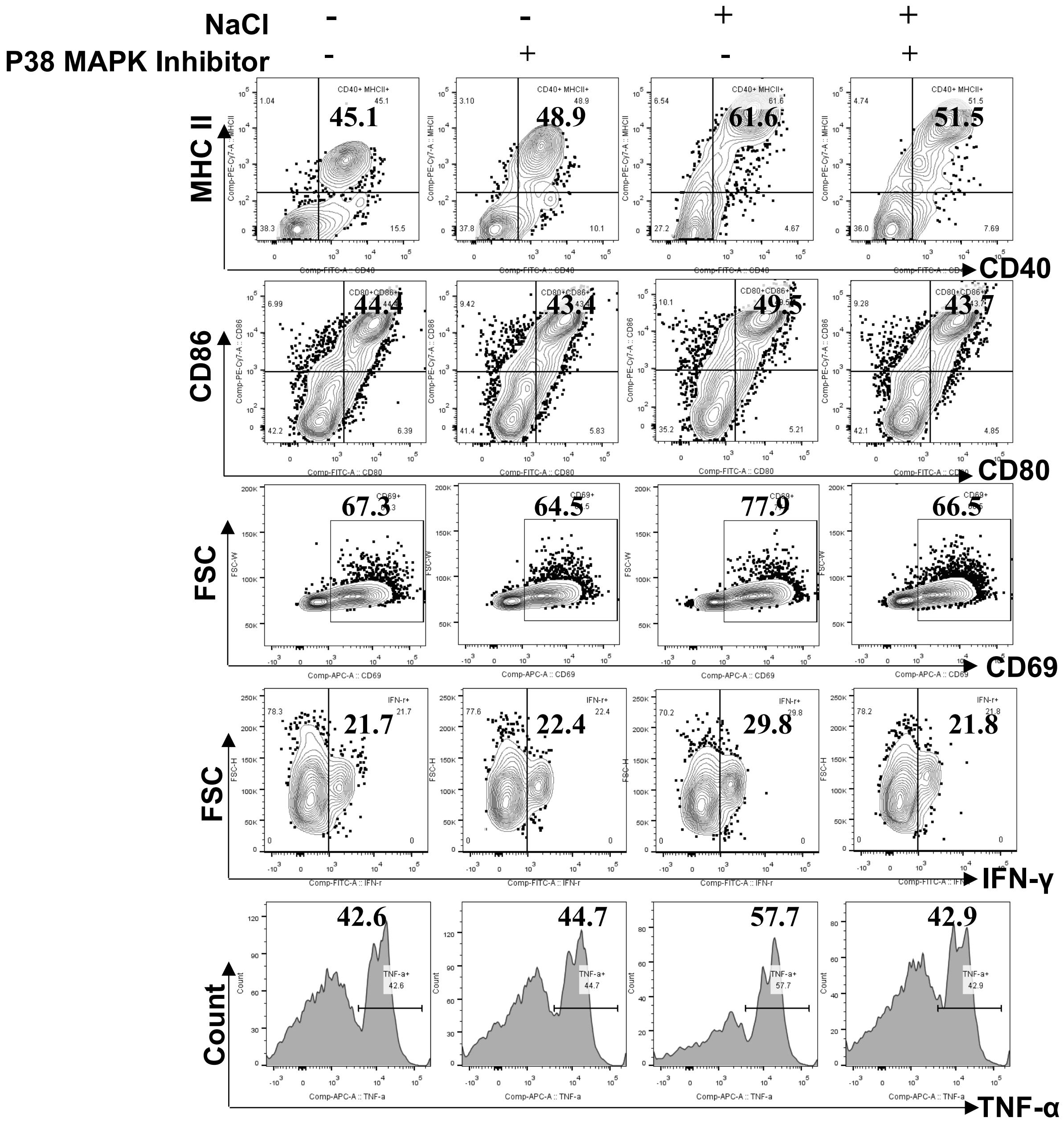

Figure S9 p38 MAPK inhibitor lessen the increased activation of NaCl on Bone morrow derived dendritic cells. Bone morrow derived dendritic cells incubated ALD-DNA with or without treatment of NaCl(20mM) were treated with p38 MAPK inhibitor (5 μM) or control (DMSO) for 24hours. Flow cytometry analyzed the expression of MHC II, CD40, CD86, CD80, CD69, IFN-γ and TNF-α of BMDCs.

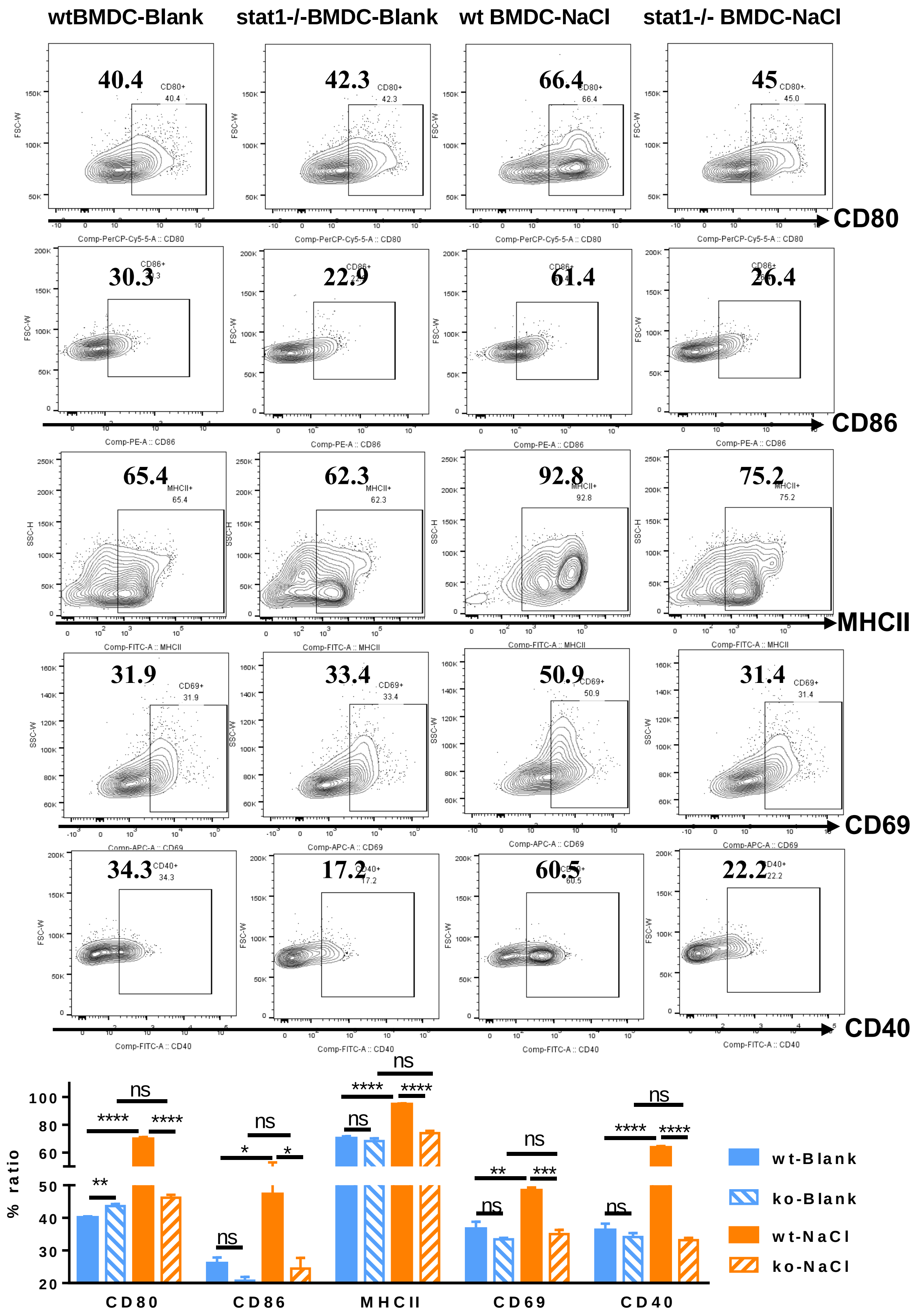

Figure S10. STAT1 depletion abated the increased activation of NaCl on Bone morrow derived dendritic cells. Bone morrow derived dendritic cells from wide type or STAT1 knock out mice were incubated ALD-DNA with or without treatment of NaCl(20mM) for 24 hours and the activation or maturation markers were examined using flow cytometry.

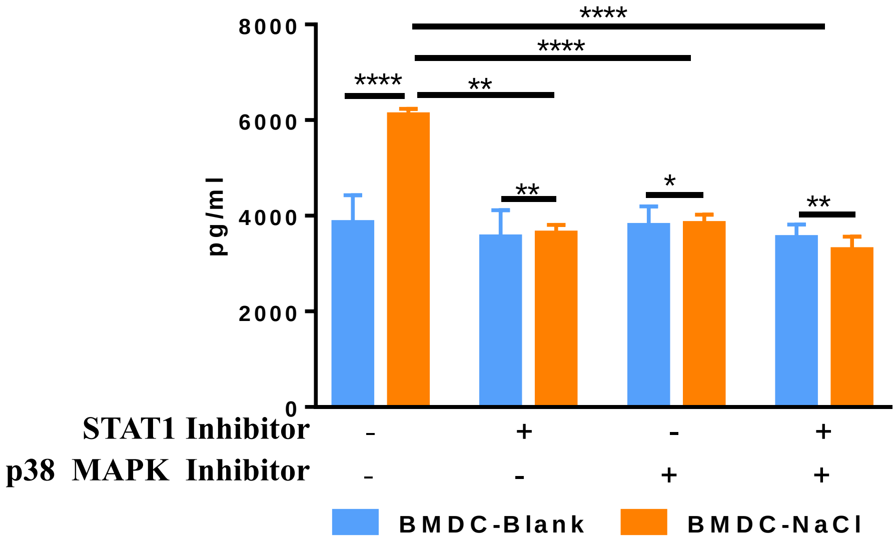

Figure S11. STAT1 and p38 MAPK inhibitor lessen the increased antigen presenting ability of NaCl on Bone morrow derived dendritic cells. Bone marrow derived dendritic cells from C57BL/6 mice were pretreated with 10 ng/ml LPS and/or 20 mM NaCl and/or 2 μg/ml STAT1 inhibitor and/or 5 μM p38 MAPK inhibitor for 24h and then collected to coculture with CFSE labeled T cells from Balb/c mice for 72 hours, ELISA quantitative of **IL-17a** in the supernatants after the DC-T co-culture for 72 hours. The results are displayed as the mean ± s.e.m. from three independent experiments. ns means no significance, $^{**}p < 0.005$, $^{***}p < 0.0005$, $^{****} < 0.0001$ using paired Student's T-tests or non-parametric test Mann-Whitney tests.

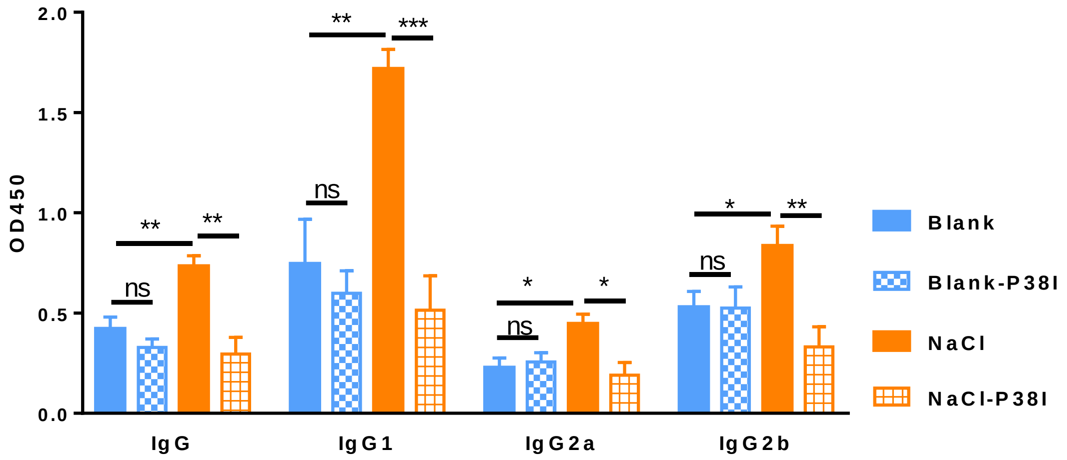

Figure S12. p38 MAPK inhibitor pretreated with BMDC decreased the anti-dsDNA antibody level from BMDC-NaCl-ALD-DNA induced lupus mice. Bone morrow derived dendritic cells incubated ALD-DNA with or without treatment of NaCl (20mM) in the presence or absence of 5 μM p38 MAPK inhibitor for 24 h and were then transferred to normal C57BL/6 mouse (n=5 in each individual experiment). ELISA experiment semi-quantitative of total IgG against ds-DNA in sera from each group lupus mice. The results are displayed as the mean ± s.e.m. from three independent experiments. ns means no significance, $**p < 0.005$, $***p < 0.0005$, $**** < 0.0001$ using paired Student's T-tests or non-parametric test Mann-Whitney tests.
